# Telemetric intracranial pressure monitoring in patients with hydrocephalus: a systematic literature review

**DOI:** 10.3389/fped.2025.1632216

**Published:** 2025-10-10

**Authors:** Kivanc Yangi, Daniel W. Gulick, Carlos E. Calderon Valero, Egemen Gok, Michael C. D'Saachs, Ishaan Bassi, Thomas J. On, Mark C. Preul

**Affiliations:** ^1^ The Loyal and Edith Davis Neurosurgical Research Laboratory, Department of Neurosurgery, Barrow Neurological Institute, St. Joseph’s Hospital and Medical Center, Phoenix, AZ, United States; ^2^ School of Electrical, Computer, and Energy Engineering, Arizona State University, Tempe, AZ, United States; ^3^ School of Biological and Health Systems Engineering, Arizona State University, Tempe, AZ, United States

**Keywords:** hydrocephalus, intracranial pressure, intracranial pressure monitoring, intracranial pressure sensor, shunt valve, telemetry

## Abstract

**Objective:**

This systematic literature review sought to examine telemetric intracranial pressure (ICP) monitoring devices, evaluate their operating principles and applications in hydrocephalus management, and highlight their advantages over traditional ICP monitoring methods.

**Materials and methods:**

A comprehensive search using Medical Subject Headings terms was conducted in the Medline (via PubMed), Scopus, and Embase databases for articles published in English. In accordance with the Preferred Reporting Items for Systematic Reviews and Meta-Analyses guidelines, a strict selection process was followed. Three reviewers independently examined the full texts of the selected articles.

**Results:**

A total of 300 articles were retrieved, with 52 meeting the inclusion criteria after removing duplicates and noneligible studies. Telemetric ICP monitoring has been studied since the 1980s, but research remained limited until 2011 (16 [31%] studies), although it increased significantly thereafter (36 [69%] studies). The Raumedic Neurovent-P-tel was introduced in 2009, and 22 of the 36 studies published since 2011 focused solely on Raumedic devices. Likewise, after the Miethke Sensor Reservoir was released in 2015, interest in this field grew, with 9 studies evaluating Miethke devices between 2017 and 2024. Since 2019, 4 studies have reported experiences using both Raumedic and Miethke devices. Among the total of 52 studies, 11 (21%) focused on pediatric patients, 10 (19%) focused on adults, 22 (42%) included both age groups, and 9 (17%) did not specify patient age.

**Conclusions:**

Telemetric ICP monitoring has emerged as a valuable tool in managing hydrocephalus, offering continuous, noninvasive monitoring that enhances diagnostic accuracy and treatment adjustments. This review highlights the increasing adoption of these devices and their potential to improve clinical outcomes while reducing hospital admissions and invasive interventions. Despite challenges such as high initial costs and sensor drift, technological advancements and further research could enhance their reliability and expand their applications in neurosurgery.

## 1 Introduction

Hydrocephalus is a neurological condition characterized by the accumulation of cerebrospinal fluid (CSF) within the cerebral ventricular system (1). The global prevalence of hydrocephalus is estimated at approximately 85 cases per 100,000 individuals, though this figure varies substantially across age groups. In children, the prevalence is around 88 per 100,000, while it drops to approximately 11 per 100,000 in the adult population. In contrast, the elderly population exhibits a markedly higher prevalence, reaching nearly 175 per 100,000, and exceeding 400 per 100,000 among individuals over the age of 80, largely due to the increased incidence of normal pressure hydrocephalus (NPH) in later life (2).

Hydrocephalus pathogenesis is complex and multifactorial, involving disturbances in CSF flow, absorption, or, in rarer cases, overproduction. Obstruction of CSF pathways, impaired absorption into the venous system, or excessive CSF secretion can all contribute to the development of hydrocephalus (2). In 1913, Walter Dandy introduced the fundamental classification of hydrocephalus into communicating and noncommunicating (obstructive) types; however, various alternative classification systems have since emerged (3, 4). For clinical clarity, adult hydrocephalus is often categorized into four main subtypes: obstructive, communicating, hypersecretory, and NPH (2). In pediatric populations, congenital or developmental hydrocephalus is more commonly encountered.

Notably, even with treatment, hydrocephalus carries a mortality rate of up to 3% in children, highlighting the critical importance of early diagnosis and appropriate intervention (5). Despite advances in endoscopic techniques such as endoscopic third ventriculostomy (ETV) and choroid plexus cauterization, ventriculoperitoneal shunting remains the first-line surgical treatment in most cases (2). However, although shunt placement remains the mainstay of surgical treatment for hydrocephalus, it is not without complications. In pediatric patients, the event-free survival rate following ventricular shunt placement is reported to be approximately 70% at 1 year, dropping to around 40% at 10 years (5). Additionally, shunt infection rates, which correlate with longer follow-up durations, can reach 15%–30%, depending on patient and procedural factors. Achieving shunt independence has been documented in only 3%–9% of cases, although reported rates vary widely across the literature due to heterogeneity in study populations and definitions (5). Given the considerable risk of shunt failure or the need for revision surgery, close postoperative surveillance is essential.

Shunt systems divert excess CSF from the brain to other body parts, such as the peritoneum, helping to regulate intracranial pressure (ICP) (6–8). However, monitoring the ICP and determining the correct valve adjustments to prevent CSF under-drainage or over-drainage can be challenging (9). Furthermore, after shunt surgery, assessing shunt dysfunction, ICP elevation, and the potential role of ICP in the patient's clinical condition is crucial. This process can be time-consuming and lead to unnecessary diagnostic tests and imaging. Most centers rely on patient-reported symptoms, which can be subtle or absent. Symptoms like headaches may not be adequately expressed or verbalized, particularly in specific patient populations, such as infants or those with limited communication abilities. Additionally, reliance on repeated computed tomography (CT) increases the frequency of outpatient visits, raises healthcare costs, and exposes infants to unnecessary ionizing radiation (10, 11).

Consequently, ICP monitoring has become a valuable diagnostic parameter for CSF disorders. Nonetheless, most methods of ICP monitoring are invasive or necessitate hospitalization, which increases the utilization of hospital resources (12). To address this issue, telemetric (wireless) sensors have emerged as safe, accurate, and cost-effective ICP monitoring tools. Although Mackay first proposed the concept of ICP monitoring with telemetric devices in 1961 (13), and the initial 2 prototypes were documented in the literature in 1967 (14, 15), the popularity of these devices significantly increased with the introduction of the Neurovent-P-tel telemetric device (Raumedic AG, Helmbrechts, Germany) in 2009. This device could accurately measure ICP with negligible zero-point drift and provide long-term performance (16–18). With technological advancements and the introduction of other telemetric devices to the market, such as sensor reservoirs, telemetric sensors present a promising alternative, offering the potential for real-time, noninvasive ICP monitoring in patients with hydrocephalus and other CSF pathologies.

We systematically reviewed the current literature on the use of telemetric sensors to measure ICP in patients with hydrocephalus. The study aimed to examine telemetric ICP monitoring devices, assess their operational principles and clinical utility, compare them with traditional methods, and identify current limitations and future research directions.

## 2 Materials and methods

### 2.1 Search strategy

Systematic searches were performed in the Medline (via PubMed), Scopus, and Embase databases, screening articles from inception to March 18, 2025, using the following keywords: [(Intracranial Pressure) OR (Intracranial Pressures) OR (Pressure, Intracranial) OR (Pressures, Intracranial) OR (Subarachnoid Pressure) OR (Pressures, Subarachnoid) OR (Pressure, Subarachnoid) OR (Subarachnoid Pressures) OR (Intracerebral Pressure) OR (Intracerebral Pressures) OR (Pressure, Intracerebral) OR (Pressures, Intracerebral)] AND [(Telemetric monitoring) OR (Telemetric) OR (Telemetrics) OR (Telemetries) OR (Telemetry) OR (Telemeter) OR (Telesensors) OR (Telemetric sensor) OR (Teletransducer) OR (Telesensor)] AND [(Hydrocephalus) OR (Hydrocephaly) OR (Communicating Hydrocephalus) OR (Hydrocephalus, Communicating) OR (Congenital Hydrocephalus) OR (Hydrocephalus, Congenital) OR (Obstructive Hydrocephalus) OR (Hydrocephalus, Obstructive) OR (Post-Traumatic Hydrocephalus) OR (Hydrocephalus, Post-Traumatic) OR (Post Traumatic Hydrocephalus) OR (Hydrocephalus Ex-Vacuo) OR (Hydrocephalus Ex Vacuo) OR (Hydrocephalus Ex-Vacuos) OR (Aqueductal Stenosis) OR (Aqueductal Stenoses) OR (Stenoses, Aqueductal) OR (Stenosis, Aqueductal) OR (Cerebral Ventriculomegaly) OR (Cerebral Ventriculomegalies) OR (Ventriculomegalies, Cerebral) OR (Ventriculomegaly, Cerebral) OR (Fetal Cerebral Ventriculomegaly) OR (Cerebral Ventriculomegalies, Fetal) OR (Cerebral Ventriculomegaly, Fetal) OR (Fetal Cerebral Ventriculomegalies) OR (Ventriculomegalies, Fetal Cerebral) OR (Ventriculomegaly, Fetal Cerebral)]. The Boolean operators “AND” and “OR” linked these Medical Subject Heading terms, ensuring maximum comprehensiveness.

### 2.2 Eligibility criteria

Our inclusion criteria focused on original research articles using telemetric devices for ICP measurement in patients with hydrocephalus. Articles were excluded if they lacked all 3 of the following key components: telemetric devices, ICP measurement, and application in patients with hydrocephalus. Review articles, errata, retracted papers, editorials, duplicate publications, and studies without accessible full texts or those not available in English were also excluded.

### 2.3 Study selection

Three independent reviewers (D.W.G., C.E.C.V., K.Y.) conducted the article screening process, considering only English-language articles. Duplicates were removed, and a strict selection procedure was followed in alignment with the Preferred Reporting Items for Systematic Reviews and Meta-Analysis (PRISMA) guidelines (19). Additionally, the reference lists of all included articles were reviewed by 2 independent reviewers (C.E.C.V., K.Y.), as recommended by systematic review guidelines (20). Disagreements during the screening were resolved through discussion, and a consensus was reached among all reviewers (D.W.G., C.E.C.V., K.Y.) to include 52 articles in the study.

## 3 Results

**Figure 1:**
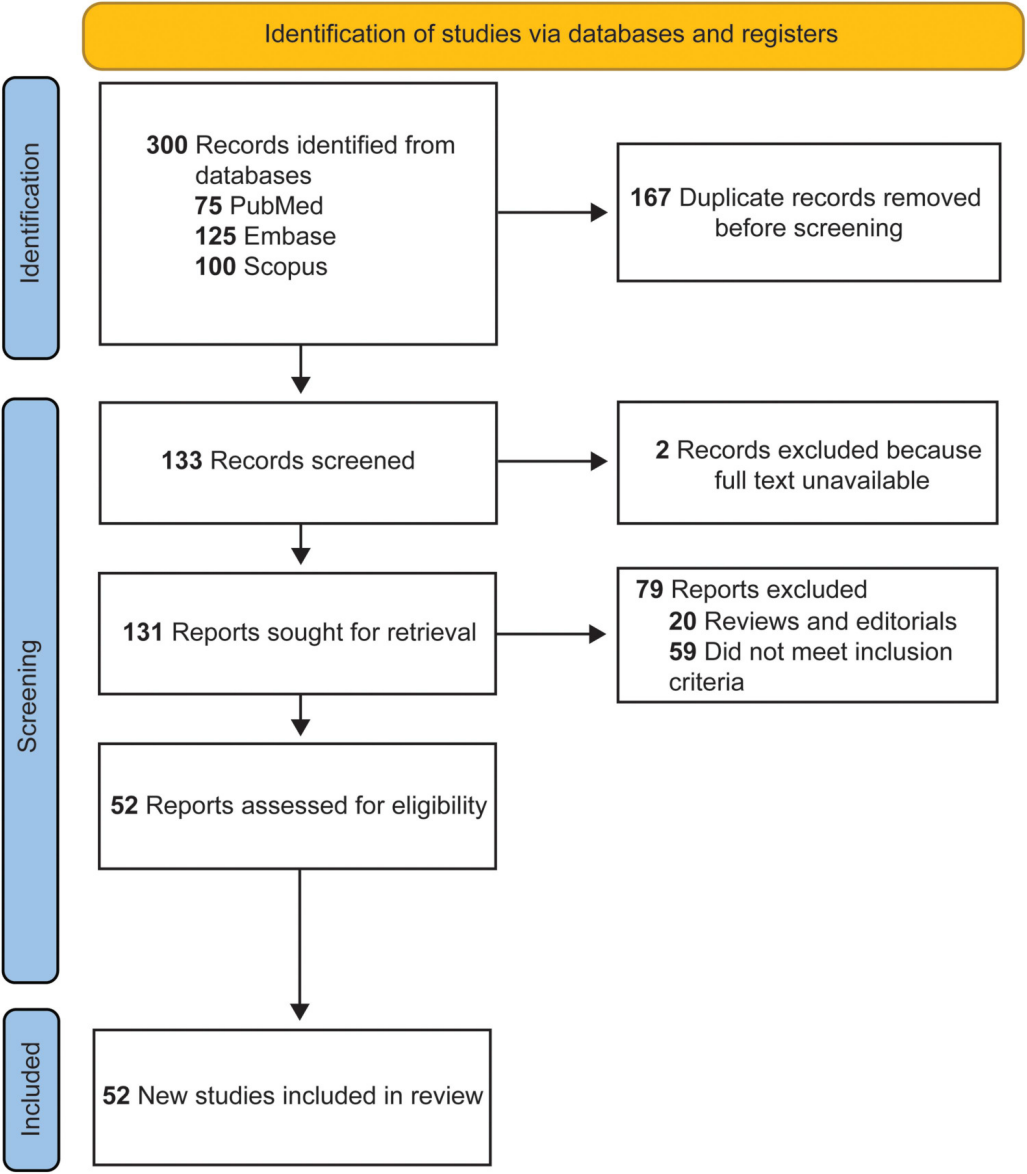
Flow diagram documenting the study selection process. Used with permission from Barrow Neurological Institute, Phoenix, Arizona.

A total of 300 articles were retrieved in the initial search. After removing 167 duplicate papers, 14 review articles, 6 editorials, and 2 articles with inaccessible full texts, 111 articles remained. Among these, 59 articles were excluded for not meeting the inclusion criteria. Upon completion of the screening, 52 articles were identified as eligible and included in the study (Table 1) (16, 21–71). The selection process, which adhered to the PRISMA guidelines, is shown in Figure 1.

**Table 1:** Studies using telemetric ICP measurement devices in the management of patients with hydrocephalus.

Study, year	Type of telemetric ICP sensor	Population	Goal
Pedersen et al. (2024) (21)	Neurovent-P-tel (Raumedic AG, Helmbrechts, Germany) and Miethke Sensor Reservoir/M.scio (Christoph Miethke GmbH & Co KG, Potsdam, Germany)	Pediatric	Evaluation of family and patient perceptions of telemetric ICP monitoring
Pandit et al. (2024) (22)	Miethke Sensor Reservoir/M.scio (Christoph Miethke GmbH & Co KG, Potsdam, Germany)	Adult	Evaluation of whether telesensor use is associated with differences in service and financial demands compared with nontelemetric reservoirs
Hornshøj Pedersen et al. (2024) (23)	Neurovent-P-tel (Raumedic AG, Helmbrechts, Germany)	Pediatric	Systematic investigation of patient and parent perceptions of the utility of telemetric ICP systems, as well as hospital contact history, to evaluate potential costs and benefits of telemetric ICP monitoring in pediatric patients with CSF disorders
Jirlow et al. (2023) (24)	Miethke Sensor Reservoir/M.scio (Christoph Miethke GmbH & Co KG, Potsdam, Germany)	Adult	Examination of the role of telemetric ICP monitoring in aiding the assessment of shunt function and necessary adjustments
Pennacchietti et al. (2023) (25)	Miethke Sensor Reservoir (Christoph Miethke GmbH & Co KG, Potsdam, Germany)	Adult and pediatric	Evaluation and definition of a maneuver protocol and its related ICP changes in an outpatient setting
Banks et al. (2022) (26)	Miethke Sensor Reservoir/M.scio (Christoph Miethke GmbH & Co KG, Potsdam, Germany)	Unspecified	Characterization of telesensor cost-effectiveness and impact on service demand
Korfias et al. (2021) (27)	Neurovent-P-tel (Raumedic AG, Helmbrechts, Germany)	Adult	Demonstration that ICP telemetry enables long-term ICP recordings, reduces hospitalization duration, and lowers overall healthcare costs
Banks et al. (2021) (28)	Miethke Sensor Reservoir/M.scio (Christoph Miethke GmbH & Co KG, Potsdam, Germany)	Unspecified	Investigation of the impact of telesensor implantation on reducing service demand and achieving net financial savings
Khan et al. (2021) (29)	A noninvasive ICP measuring reservoir	Adult	Investigation of the utility of postneuroendoscopy ICP monitoring using a new-generation, noninvasive ICP measuring reservoir
Kommer et al. (2021) (30)	Raumedic telemetric ICP probe (details not available)	Adult and pediatric	Determination of whether short recordings were reflective of longer periods of monitoring and assessment of the safety of one-off measurement
Rot et al. (2020) (31)	Neurovent-P-tel (Raumedic AG, Helmbrechts, Germany) and Miethke Sensor Reservoir (Christoph Miethke GmbH & Co KG, Potsdam, Germany)	Adult	Analysis of the differences in ICP values measured with Neurovent-P-tel probe, Miethke Sensor Reservoir, and EVD
Pennacchietti et al. (2020) (32)	Neurovent-P-tel (Raumedic AG, Helmbrechts, Germany) and Miethke Sensor Reservoir (Christoph Miethke GmbH & Co KG, Potsdam, Germany)	Adult and pediatric	Evaluation of the effectiveness of telemetric ICP measurement in enhancing the clinical management of shunted patients with complex hydrocephalus
Pedersen et al. (2020) (33)	Neurovent-P-tel (Raumedic AG, Helmbrechts, Germany)	Pediatric	Evaluation of the experience of using long-term telemetric ICP monitoring in pediatric patients
Müller et al. (2019) (34)	Neurovent-P-tel (Raumedic AG, Helmbrechts, Germany)	Adult	Proposal of a maneuver for outpatient telemetric ICP recording and evaluation of its test-retest reliability
Norager et al. (2019) (35)	Neurovent-P-tel (Raumedic AG, Helmbrechts, Germany) and Miethke Sensor Reservoir (Christoph Miethke GmbH & Co KG, Potsdam, Germany)	Adult and pediatric	Identification of appropriate uses of Neurovent-P-tel and Miethke Sensor Reservoir
Tschan et al. (2019) (36)	Neurovent-P-tel (Raumedic AG, Helmbrechts, Germany)	Adult and pediatric	Report on a new home setup for telemonitoring ICP and assess the usefulness of these devices, particularly for patients living far from hospitals
Norager et al. (2018) (37)	Neurovent-P-tel (Raumedic AG, Helmbrechts, Germany)	Adult and pediatric	Investigation of the clinical performance, technical durability, survival time, and drift of the telemetric ICP sensor
Antes et al. (2018) (38)	Miethke Sensor Reservoir (Christoph Miethke GmbH & Co KG, Potsdam, Germany)	Adult and pediatric	Evaluation of the telemetric device for individually adjusting shunt valves on the basis of ICP measurements
Thompson et al. (2018) (39)	Miethke Sensor Reservoir (Christoph Miethke GmbH & Co KG, Potsdam, Germany)	Unspecified	Assessment of the financial benefits of telemetric device implantation
Thompson et al. (2018) (40)	Miethke Sensor Reservoir (Christoph Miethke GmbH & Co KG, Potsdam, Germany)	Unspecified	Evaluation of the reliability of the telemetric device in managing patients with complex hydrocephalus
Ertl et al. (2017) (41)	Aesculap-Miethke Sensor Reservoir (Christoph Miethke GmbH & Co KG, Potsdam, Germany)	Adult	Evaluation of the usefulness of the sensor reservoir in reliably measuring ICP changes in patients with a shunt system
Barber et al. (2017) (42)	Neurovent-P-tel (Raumedic AG, Helmbrechts, Germany)	Pediatric	Examination of the overall cost of telemetric device implantation and its impact on reducing hospital admissions, and collection and analysis of patient and family feedback
Antes et al. (2016) (16)	Neurovent-P-tel (Raumedic AG, Helmbrechts, Germany)	Adult and pediatric	Examination of technical aspects, handling, possibilities of data analysis, and efficiency of the telemetric probe in clinical routine
Andresen et al. (2016) (43)	Neurovent-P or Neurovent-P-tel intraparenchymal probes (Raumedic AG, Helmbrechts, Germany)	Adult and pediatric	Quantification of the effects of postural changes on ICP in healthy and ill subjects
Maeske et al. (2016) (44)	Neurovent-P-tel (Raumedic AG, Helmbrechts, Germany)	Adult and pediatric	Examination of ICP measurements in mobile patients in their everyday environment
Andresen et al. (2015) (47)	Neurovent-P or Neurovent-P-tel (Raumedic AG, Helmbrechts, Germany)	Adult and pediatric	Investigation of ICP in different body postures in both healthy and ill subjects
Farahmand et al. (2015) (45)	Intraparenchymatous ICP sensor (Raumedic AG, Helmbrechts, Germany)	Adult	Analysis of the ICP and ICP wave amplitude at different shunt settings and body positions in patients with hydrocephalus
Raffalli-Ebezant et al. (2014) (46)	Neurovent-P-tel (Raumedic AG, Helmbrechts, Germany)	Pediatric	Evaluation of the management, cost analysis, and early clinical outcomes of telemetric ICP monitor use
Antes et al. (2014) (48)	Neurovent-P-tel (Raumedic AG, Helmbrechts, Germany)	Adult and pediatric	Providing sufficient ICP data for long-term observation and early detection of endoscopy failures and complications
Lilja et al. (2014) (49)	Neurovent-P-tel (Raumedic AG, Helmbrechts, Germany)	Adult and pediatric	Evaluation of the clinical utility of long-term telemetric ICP monitoring and identification of its advantages and challenges to guide future improvements in the technology
Freimann et al. (2014) (50)	Neurovent-P-tel (Raumedic AG, Helmbrechts, Germany)	Adult and pediatric	Presentation of first experiences with telemetric ICP-guided valve adjustments in cases with the combination of an adjustable differential pressure valve and adjustable gravitational unit
Antes et al. (2014) (51)	Neurovent-P-tel (Raumedic AG, Helmbrechts, Germany)	Adult and pediatric	Evaluation of clinical and radiological findings after insertion of an intraparenchymal telemetric ICP monitor
Tschan et al. (2013) (52)	Neurovent-P-tel (Raumedic AG, Helmbrechts, Germany)	Pediatric	Presentation of first long-term experience with the telemetric intraparenchymal probe in children
Elixmann et al. (2012) (53)	Neurovent-P-tel (Raumedic AG, Helmbrechts, Germany)	Adult	Provide insight into the differential pressure across a hydrocephalus valve in everyday life
Welschehold et al. (2012) (54)	Neurovent-P-tel (Raumedic AG, Helmbrechts, Germany)	Adult and pediatric	Presentation of first clinical experiences with a new telemetric ICP monitoring device
Tschan et al. (2011) (55)	Neurovent-P-tel (Raumedic AG, Helmbrechts, Germany)	Adult and pediatric	Presentation of experience with a new telemetric intraparenchymal pressure probe, with the transducer placed over the calvaria and beneath the galea
Frim and Lathrop (2000) (56)	TeleSensor (Radionics, Inc, Burlington, MA, USA)	Adult and pediatric	Assessment of the *in vivo* impact of variable pressure valve adjustments on ICP changes using a noninvasive telemonitor, serving as an alternative to radiographic confirmation and a method for validating ICP changes
Frim and Goumnervoa (2000) (57)	TeleSensor (models ICP-M4 and ICP-M3) (Radionics, Inc, Burlington, MA, USA)	Adult and pediatric	Integration of telemonitoring devices with various shunt systems to evaluate the performance characteristics of these valve systems in relation to IVP at different head elevation levels
Richard et al. (1999) (58)	A custom ICP sensor designed by Telemeasurement GmbH, Aachen, Germany	Adult and pediatric	Highlighting the advantages of telesensors, including their improved assessment of shunt dysfunction and detection of marginal CSF pressure increases, alongside their practicality, simplicity in outpatient measurements, and potential to reduce the reliance on costly CT or MRI
Wayenberg (1998) (59)	RTT (Rotterdam, Netherlands)	Pediatric	Obtaining accurate information about ICP and changes in cerebral compliance across a wide range of clinical conditions without relying on invasive techniques
Munshi et al. (1998) (60)	TeleSensor (Radionics, Inc, Burlington, MA, USA)	Adult and pediatric	Examination of the *in vivo* IVP dynamics of ventriculopleural shunts using a commercially available implantable telemonitor
Miyake et al. (1997) (71)	Osaka telesensor (Nagano Keiki Seisakusyo Co Ltd, Tokyo, Japan)	Unspecified	Investigation of the clinical usefulness of new ventriculoperitoneal shunting with an Osaka telesensor
Frim and Goumnervoa (1997) (61)	TeleSensor device (Radionics, Inc, Burlington, MA, USA)	Adult	Examination and documentation of IVP dynamics in an adult after endoscopic third ventriculocisternostomy performed as treatment for hydrocephalus associated with aqueductal stenosis
Longatti and Carteri (1994) (70)	Telemetric ICP detector (details not available)	Unspecified	Measurement of acute changes in ICP after temporarily closing the shunt valve to determine whether the shunt remained necessary
Wayenberg et al. (1993) (62)	RTT (Rotterdam, Netherlands)	Pediatric	Demonstration that the RTT provided accurate and reproducible measurements of ICP
Kamiryo et al. (1991) (63)	NS10 (Nagano Keiki Seisakusho, Japan)	Pediatric	Report of a case of hydrocephalus showing slit ventricle syndrome after multiple shunt revisions treated with a programmable pressure valve; ICP was monitored with a telemetric sensor
Yamaguchi et al. (1990) (64)	Telemetric ICP sensor (details not available)	Unspecified	Measurement of ventricular fluid pressure in 10 hydrocephalic patients before and after ventriculoperitoneal shunts
Chapman et al. (1990) (65)	TeleSensor (Radionics, Inc, Burlington, MA, USA)	Adult and pediatric	Description of the disruption of postural IVP regulation caused by shunt placement and the impact of different shunt systems and antisiphon devices on this issue
Güçer et al. (1988) (66)	A permanently implanted epidural sensor	Unspecified	Discussion of the stability, safety, and accuracy of the sensor, along with the causes of drift and potential solutions, and presentation of a comparison with other epidural telemetric monitoring systems
Maas and de Jong (1986) (67)	RTT (Rotterdam, Netherlands)	Pediatric	Presentation of the problems encountered in the development of the device and report on results obtained with the RTT in clinical use
Chapman et al. (1984) (69)	Custom telemetric monitoring system	Unspecified	Proposition of an alternative to EVD (telemetric ICP monitoring) for the management of postoperative hydrocephalus
Nulsen et al. (1980) (68)	Custom telemetric device	Pediatric	Description of the capacity to monitor ICP accurately by telemetry

CSF, cerebrospinal fluid; CT, computed tomography; EVD, external ventricular drain; ICP, intracranial pressure; IVP, intraventricular pressure; MRI, magnetic resonance imaging; RTT, Rotterdam Teletransducer.

**Figure 2:**
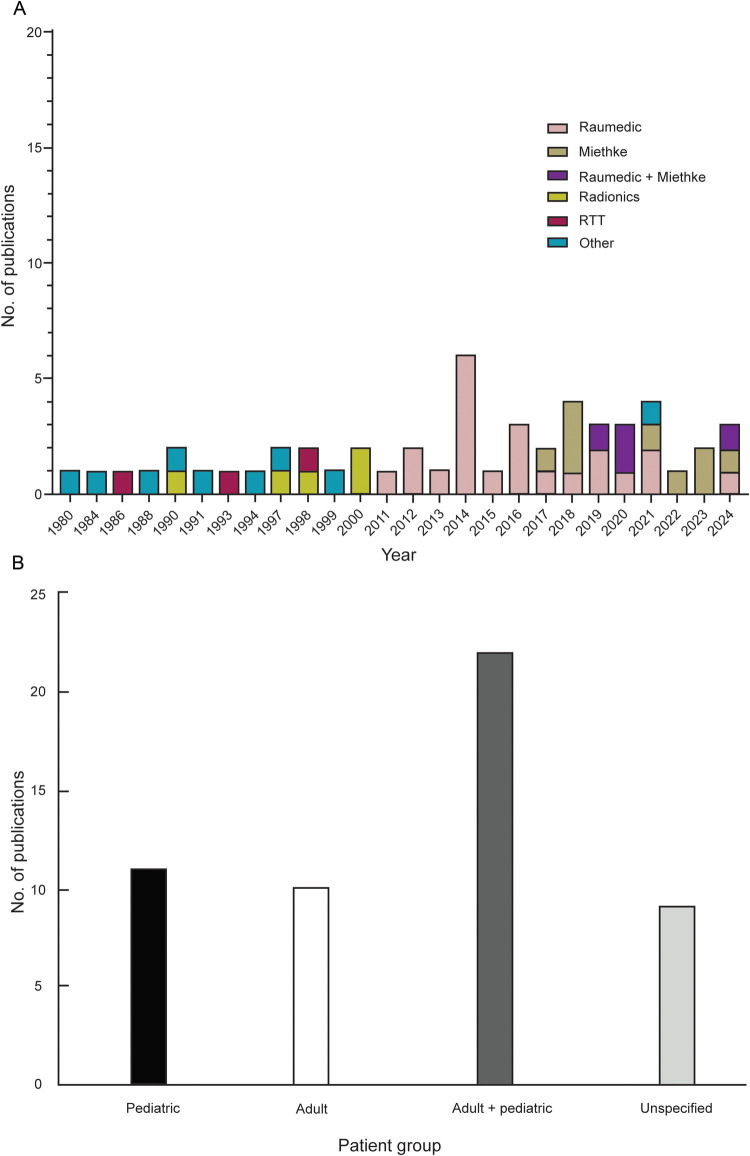
**(A)** Distribution of 52 studies describing the use of telemetric devices for intracranial pressure measurement in the management of patients with hydrocephalus by year and brand. **(B)** Distribution of the telemetric studies by patient population (pediatric, *n* = 11; adult, *n* = 10; adult and pediatric, *n* = 22; unspecified, *n* = 9). Miethke, Miethke Sensor Reservoir; Radionics, Radionics TeleSensor; Raumedic, Raumedic Neurovent-P-tel; RTT, Rotterdam Teletransducer. Used with permission from Barrow Neurological Institute, Phoenix, Arizona.

Although the role of telemetric ICP monitoring devices in managing patients with hydrocephalus has been studied since 1980, we found that there were relatively few publications before 2011 (16 of 52, 31%), with earlier studies mainly involving devices such as the Rotterdam Teletransducer (RTT) (Erasmus University, Rotterdam, Netherlands) (*n* = 3), Radionics TeleSensor (Radionics, Inc, Burlington, MA, USA), (*n* = 5), and other telemetry-based sensors (*n* = 8). However, from 2011 onward, there was a notable and steady increase in research on telemetric ICP devices each year (36 of 52, 69%) (Figure 2).

Following the introduction of the Neurovent-P-tel in 2009, there was a notable increase in related publications, with Raumedic telemetric devices examined in 22 of the 36 studies published since 2011. Similarly, the number of publications increased after the introduction of the Miethke Sensor Reservoir (Christoph Miethke GmbH & Co KG, Potsdam, Germany) in 2015, with many studies beginning to evaluate the effectiveness of the Miethke devices (*n* = 9). Since 2019, several centers have reported their experiences using Raumedic and Miethke devices (*n* = 4). On the other hand, since 2011, only 1 study has examined telemetric devices other than Raumedic or Miethke (Figure 2).

Among 52 studies that used telemetric devices for ICP measurement in managing patients with hydrocephalus, 11 (21%) focused exclusively on pediatric patients, 10 (19%) focused on adult patients, and 22 (42%) included both adult and pediatric patient groups. In 9 (17%) of the studies, the age groups of the included patients were not specified.

**Figure 3:**
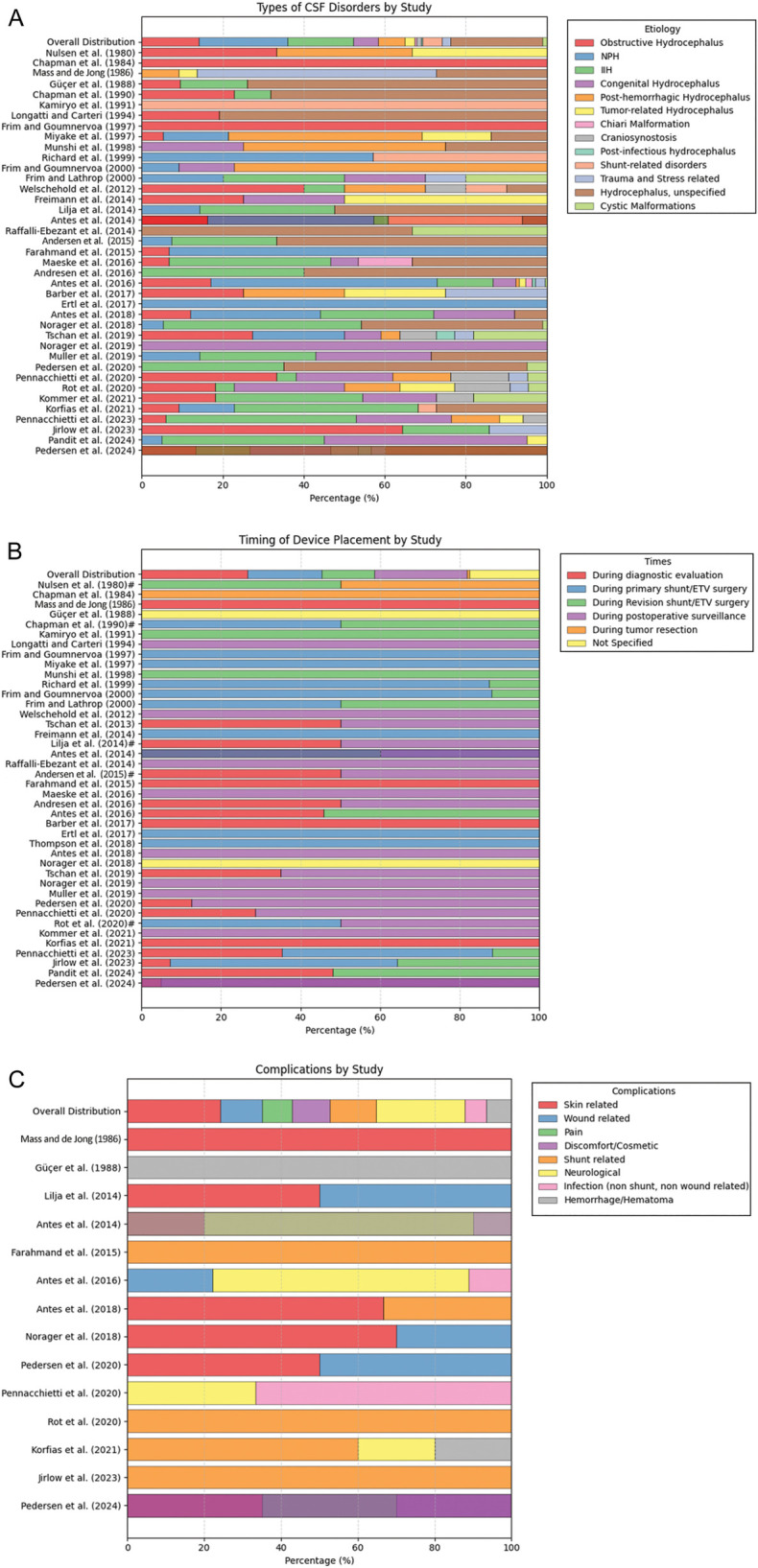
Summary of cerebrospinal fluid (CSF) disorder types, device placement timing, and complication rates as reported across the reviewed studies. **(A)** Types of CSF disorders. **(B)** Timing of device insertion. In cases in which the number of devices was not specified but only a single “timing of device placement” was mentioned, 100% of the corresponding bar was allocated to that indication. Conversely, when multiple indications were reported without exact counts, the bar was proportionally divided equally among them. Studies with such assumptions are denoted with a hash symbol (#). **(C)** Complications. Studies lacking complication data, either due to the absence of reported events or omission of relevant information, were excluded from this analysis. The bar graph represents proportional distributions derived exclusively from the reported numbers. Used with permission from Barrow Neurological Institute, Phoenix, Arizona.

Furthermore, to ensure clinical correlation, the most clinically relevant studies were examined (Table 2). Among the 42 clinically relevant studies published between 1980 and 2024, the pooled mean (SD) patient age was 44.03 (23.98) years. Of these studies, 8 focused exclusively on pediatric patients, 7 on adults, 23 included both age groups, and 4 did not specify the patient population. In the overall distribution of etiologies reported across the included studies, the most frequently observed categories were unspecified hydrocephalus (*n* = 281), NPH (*n* = 274), and idiopathic intracranial hypertension (*n* = 202). These were followed by obstructive hydrocephalus (*n* = 175), posthemorrhagic hydrocephalus (*n* = 81), congenital hydrocephalus (*n* = 76), and shunt-related disorders (*n* = 61). Less commonly reported etiologies included tumor-related hydrocephalus (*n* = 31), trauma- or stress-associated hydrocephalus (*n* = 27), cystic malformations (*n* = 14), craniosynostosis (*n* = 13), Chiari malformation (*n* = 6), and postinfectious hydrocephalus (*n* = 4) (Figure 3).

**Table 2:** Summary of clinically relevant studies on telemetric ICP monitoring (1980–2024): population, CSF disorder types, device details, and outcomes.

Study	Population	Age, yrs	No. of pts, by type of CSF disorder	Timing of device placement	Device(s) used	Functional duration of the device	Follow-up duration	Shunt or device revision	Complication(s)
Pedersen et al. (2024) (21)	Pediatric: 16^a^	<4: 1 pt; 4–8: 4 pts; 9–12: 4 pts; 13–16: 3 pts; unspecified: 4 pts	Obstructive hydrocephalus: 4; trauma and stress related: 1; hydrocephalus, unspecified: 12; IIH: 4; postinfectious hydrocephalus: 1; congenital hydrocephalus: 6; Chiari malformation: 2; craniosynostosis: 2	Diagnostic evaluation: 2/41; postoperative surveillance: 39/41	Neurovent-P-tel: 40/41; Miethke SR: 1/41	Mean (range): 337.6 (12–1,290) days	NS	Device revision/removal: 32/41 (no longer functional: 17/32; patient/parent request: 1/32; pain/irritation: 4/32; minor wound defect: 1/32; liquor accumulation: 1/32; infection: 2/32; unknown: 6/32)	Skin irritation: 7; cosmetic issues: 6; pain due to sensor: 7
Pandit et al. (2024) (22)	Adult: 136 (telesensor: 74; control: 62 controls)	Mean (SD): 38.3 (14.7)	IIH: 16; congenital hydrocephalus: 20; tumor-related hydrocephalus: 2; NPH: 2^f^	Initial shunt placement: 26/48; shunt revision: 28/48	Miethke SR	NS	Minimum 2 years	Mean (SD) shunt revision count per pt: telesensor group, 0.27 (0.61); control group, 0.71 (1.64)	NS
Hornshøj Pedersen et al. (2024) (23)	Pediatric: 3	Mean (SD): 5.0 (3.36)	Hydrocephalus, unspecified: 3	Postoperative surveillance: 4/4	Neurovent-P-tel	NS	1 pt >5 years; 2 pts <1 year	Device revision: 1	Fear of teasing at school: 1; cosmetic challenges: 1; wire wrapping during sleep: 1
Jirlow et al. (2023) (24)	Adult: 14	Mean (range): 30.3 (21–50)	IIH: 3; obstructive hydrocephalus: 9; trauma and stress related: 2	Primary shunt surgery: 8/14; revision surgery: 5/14; stand-alone placement: 1/14	Miethke SR	NS	8 months to 5 years	Shunt revisions: 5	Shunt dysfunction: 4; shunt infection: 1
Pennacchietti et al. (2023) (25)	Adult and pediatric: 17	Median (range): shunt group, 15.8 (4–35.2); stand-alone group, 11.9 (3.6–17.7)	IIH: 8; congenital hydrocephalus: 4; posthemorrhagic hydrocephalus: 2; obstructive hydrocephalus: 1; tumor-related hydrocephalus: 1; craniosynostosis: 1	Primary shunt surgery: 9/17; revision surgery: 2/17; stand-alone placement: 6/17	Miethke SR	NS	NS	NS	NS
Korfias et al. (2021) (27)	Adult: 22	Mean (range): 40.8 (21–65)	IIH: 10; NPH: 3; hydrocephalus, unspecified: 6; obstructive hydrocephalus: 2; shunt-related disorders: 1	Diagnostic evaluation	Neurovent-P-tel	NS	NS	No device revision; shunt-related revisions: 3	Local cerebral edema around catheter: 1; epileptic seizure due to small hematoma after device removal: 1; shunt-related complications: 3
Kommer et al. (2021) (30)	Adult and pediatric: 11	Median (range): 14.2 (2.4–46.2)	IIH: 4; cystic malformation: 2; congenital hydrocephalus: 2; craniosynostosis: 1; obstructive hydrocephalus: 2	Postoperative surveillance	Neurovent-P-tel	NS	NA (all inpatients)	NA	No device-related complications reported
Rot et al. (2020)^b^ (31)	Adult: 17	Mean (range): 57 (26–80)	Congenital hydrocephalus: 6; posthemorrhagic hydrocephalus: 3; obstructive hydrocephalus: 4; tumor-related hydrocephalus: 3; craniosynostosis: 3; cystic malformation: 1; trauma and stress related: 1; IIH: 1	Primary shunt surgery (Neurovent-P-tel); postoperative surveillance (Miethke SR)	Neurovent-P-tel (first 3 months) and Miethke SR (after 3 months)	NS	NS	NS	Overdrainage: 1
Pennacchietti et al. (2020) (32)	Adult and pediatric: 21	Median (range): 16.5 (10–39.5)	Congenital hydrocephalus: 5; posthemorrhagic hydrocephalus: 3; obstructive hydrocephalus: 7; craniosynostosis: 3; cystic malformation: 1; IIH: 1; trauma and stress related: 1	Diagnostic evaluation: 6/21 (all Miethke SR); postoperative surveillance: 15/21 (Neurovent-P-tel: 8; Miethke SR: 7)	Neurovent-P-tel: 8/21; Miethke SR: 13/21	Median (range): 4.2 (4–13) months	NS	No device revision; explantations due to complications: 3	Neurovent-P-tel: 1 infection, 1 seizure; Miethke SR: 1 infection
Pedersen et al. (2020) (33)	Pediatric: 20	Median (range): 11 (2–18)	Hydrocephalus, unspecified: 12; IIH: 7; cystic malformation: 1	Diagnostic evaluation: 4/32; postoperative surveillance: 28/32	Neurovent-P-tel	Median (range): 523 (42–2,067) days	NS	17 devices explanted (12 replaced): complications: 2; technical defects: 12; parent request: 3	Wound infection: 1; skin erosion: 1
Müller et al. (2019) (34)	Adult: 7	Mean (SD): 48.6 (12.2)	IIH: 2; congenital hydrocephalus: 2; NPH: 1; hydrocephalus, unspecified: 2	Postoperative surveillance	Neurovent-P-tel	NS	NS	NS	NS
Norager et al. (2019) (35)	Adult and pediatric: 2	7, 27	Congenital hydrocephalus: 2	Postoperative surveillance	Neurovent-P-tel and Miethke SR	Case 1: each Neurovent-P-tel implanted for months at a time, with repeated reimplantations; case 2: same SR functioning >2 years	Case 1: 2 years; case 2: 767 days	Case 1: 3 reimplantations of Neurovent-P-tel over 2 years due to suspected measurement issues; case 2: Miethke SR was implanted due to multiple failures of Neurovent-P-tel	NS
Tschan et al. (2019) (36)	Adult and pediatric: 20	Mean (SD): 33.2 (16.3)	IIH: 5; obstructive hydrocephalus: 6; cystic malformations: 4; posthemorrhagic hydrocephalus: 1; postinfectious hydrocephalus: 1; congenital hydrocephalus: 2; trauma and stress related: 1; cystic malformations: 2	Diagnostic evaluation: 7/20; postoperative surveillance: 13/20	Neurovent-P-tel: 20	Mean (SD): 278.5 (250.1) days	NS	None	None
Norager et al. (2018) (37)	Adult and pediatric: 119	Median (IQR): 30 (17–50)	Hydrocephalus, unspecified: 42; IIH: 46; NPH: 5; cystic malformation: 1^e^	NS	Neurovent-P-tel: 125	Median (95% CI): 556 (382–605) days	NS	6 reimplantations	Skin damage (erosion): 5; wound infection: 3; ethylene oxide allergy: 2
Antes et al. (2018) (38)	Adult and pediatric: 25	Mean (SD): 53.6 (20.7)	NPH: 8; IIH: 7; congenital hydrocephalus: 5; obstructive hydrocephalus: 3; hydrocephalus, unspecified: 2	Postoperative surveillance	Miethke SR	NS	Mean (SD): 213.8 (119.4) days	No explantation/reimplantation	Wound healing disorders: 2; shunt infection: 1
Thompson et al. (2018) (39)	Age group not defined: 60	NS	NS	Shunt surgery	Miethke SR	NS	NS	NS	NS
Ertl et al. (2017) (41)	Adult: 2	66, 78	NPH: 2	Primary shunt surgery	Miethke SR	NS	NS	NS	NS
Barber et al. (2017) (42)	Pediatric: 4	Mean (SD): 9.0 (5.1)	Posthemorrhagic hydrocephalus: 1; trauma and stress related: 1; obstructive hydrocephalus: 1; tumor-related hydrocephalus: 1	Diagnostic evaluation	Neurovent-P-tel	Range: 460–632 days	NS	NS	NS
Antes et al. (2016) (16)	Adult and pediatric: 247	Mean (SD): 59.3 (20.7)	Postinfectious hydrocephalus: 2; tumor-related hydrocephalus: 4; IIH: 34; NPH: 138; obstructive hydrocephalus: 42; posthemorrhagic hydrocephalus: 2; congenital hydrocephalus: 14; Chiari malformation: 4; trauma and stress related: 6; cystic malformation: 1	Diagnostic evaluation: 105; shunt revision or prior to planned ETV: 124^c^	Neurovent-P-tel	Mean (SD): 46.9 ± 26.0 days	NS	All devices were explanted; no shunt revisions	Hemiparesis: 1; new-onset seizures: 11; brain abscess: 2; wound infection: 4
Andresen et al. (2016) (43)	Adult and pediatric: 15^i^	Mean (range): 32 (8–71)	Hydrocephalus, unspecified: 9; IIH: 6	Diagnostic evaluation or postoperative surveillance	Neurovent-P-tel	NS	NS	NS	NS
Maeske et al. (2016) (44)	Adult and pediatric: 15	Mean (SD): 29.1 (14.6)	IIH: 6; hydrocephalus, unspecified: 5; Chiari malformation: 2; obstructive hydrocephalus: 1; congenital hydrocephalus: 1	Postoperative surveillance	Neurovent-P-tel	NS	NS	NS	NS
Andresen et al. (2015) (47)	Adult and pediatric: 27	Mean (range): 35.11 (8–71)	IIH: 7; NPH: 2; hydrocephalus, unspecified: 18	Diagnostic evaluation or postoperative surveillance	Neurovent-P-tel	NS	NS	NS	NS
Farahmand et al. (2015) (45)	Adult: 15	Mean (SD): 70.5 (8.9)^d^	NPH: 14; obstructive hydrocephalus: 1	Primary shunt surgery	Neurovent-P-tel	All devices explanted within 1 day	3 months for every pt	All devices explanted within 1 day	Shunt infection: 1
Raffalli-Ebezant et al. (2014) (46)	Pediatric: 3	NS	Hydrocephalus, unspecified: 2; cystic malformation: 1	Postoperative surveillance: 3	Neurovent-P-tel	Mean: 5 days	NS	1 pt required further neurosurgical intervention after pathological ICP detection	None
Antes et al. (2014) (48)	Adult and pediatric: 24	Mean (SD): 46.9 (18.4)	Obstructive hydrocephalus: 24	Primary ETV procedure	Neurovent-P-tel	Mean: 106.1 h	NS	Proceeding with shunt due to failed ETV in 7 cases	Intraventricular bleeding: 2; thalamic contusion: 1
Lilja et al. (2014) (49)	Adult and pediatric: 21	Median (range): 28 (2–83)	Hydrocephalus, unspecified: 11; IIH: 7; NPH: 3	Diagnostic evaluation or postoperative surveillance	Neurovent-P-tel: 22^g^	Median (range): 248 (49–666) days	NS	29 of 86 recordings led to surgical shunt revision	Late wound infection: 2; ethylene oxide allergy: 2
Freimann et al. (2014) (50)	Adult and pediatric: 4	Mean (SD): 12.5 (6.0)	Tumor-related hydrocephalus: 2; congenital hydrocephalus: 1; obstructive hydrocephalus: 1	Primary shunt revision surgery: 4	Neurovent-P-tel	Mean (SD): 8.0 (5.9) months	NS	NS	None
Antes et al. (2014) (51)	Adult and pediatric: 185	Mean (SD): 54.9 (23.0)	NPH: 68; occlusive hydrocephalus: 27; IIH: 6; hydrocephalus, unspecified: 10; shunt-related disorders: 55^h^	Diagnostic evaluation: 111; postoperative surveillance: 74	Neurovent-P-tel: 185	Mean (SD): 60.7 (58.1) days	NS	23 shunt or valve revision surgeries	Brain abscess: 1; superficial wound infections: 2; new-onset seizures: 6; temporary hemiparesis: 1
Tschan et al. (2013) (52)	Pediatric: 26	Mean (range): 10.5 (1.5–18)	NS^j^	Diagnostic evaluation or postoperative surveillance	Neurovent-P-tel	Mean (range): 61 (8–209) days	NS	NS	None
Welschehold et al. (2012) (54)	Adult and pediatric: 10	Mean (SD): 21.1 (16.95)	Obstructive hydrocephalus: 4; posthemorrhagic hydrocephalus: 2; hydrocephalus, unspecified: 1; IIH: 1; craniosynostosis: 1; shunt-related disorders: 1	Postoperative surveillance: 10	Neurovent-P-tel	Mean (SD): 40.1 (51.1) days^k^	NS	Shunt revision or implantation in 3 pts	None
Frim and Lathrop (2000) (56)	Adult and pediatric: 10	Mean (range): 29.4 (1–84)	Congenital hydrocephalus: 2; NPH: 2; cystic malformation: 2; IIH: 3; trauma and stress related: 1	Primary and revision shunt placement	Radionics TeleSensor: 12	12 months	NS	2 shunt revisions due to infection	NS
Frim and Goumnervoa (2000) (57)	Adult and pediatric: 22	Median (range): 27.5 (7–71)	Congenital hydrocephalus: 3; NPH: 2; posthemorrhagic hydrocephalus: 17	Initial shunt placement: 22; revision shunt surgery: 3	Radionics TeleSensor: 25	NS	NS	3 shunt systems revised after initial pressures obtained	NS
Richard et al. (1999) (58)	Adult and pediatric: 7	Mean (SD): 40.29 (19.18)	NPH: 4; shunt-related disorders: 3	Primary shunt placement: 7; revision shunt placement: 1	Telemeasurement GmbH integrated ICP-sensor (custom made): 8	NS	14–17 months	1 shunt revision due to traumatic valve deformation	NS
Munshi et al. (1998) (60)	Adult and pediatric: 4	Range: 12–71	Posthemorrhagic hydrocephalus: 2; hydrocephalus, unspecified: 1; congenital hydrocephalus: 1	Shunt revision: 4	Radionics TeleSensor	NS	NS	NS	NS
Miyake et al. (1997) (71)	Adult and pediatric: 94	NS	Obstructive hydrocephalus: 5; tumor-related hydrocephalus: 16; NPH: 15; posthemorrhagic hydrocephalus: 45; hydrocephalus, unspecified: 13	Primary shunt surgery: 94	Osaka telesensor	Mean (range): 14.5 (1–44) months	NS	NS	NS
Frim and Goumnervoa (1997) (61)	Adult: 1	30	Obstructive hydrocephalus: 1	Primary ETV procedure: 1	Radionics TeleSensor: 1	NS	NS	None	NS
Longatti and Carteri (1994) (70)	Adult and pediatric: 21	NS	Obstructive hydrocephalus: 4; hydrocephalus, unspecified: 17	Postoperative surveillance: 21	Telemetric ICP detector	NS	NS	None	NS
Kamiryo et al. (1991) (63)	Pediatric: 1	8	Shunt-related disorders: 1	Shunt revision surgery: 1	Nagano Keiki NS20 telemetric ICP sensor	NS	NS	None	NS
Chapman et al. (1990) (65)	Adult and pediatric: 22	Mean (SD): 25.41 (18.93)	Obstructive hydrocephalus: 5; IIH: 2; hydrocephalus, unspecified: 15	Primary and revision shunt placement	Radionics TeleSensor	NS (up to 4 years in some pts)	NS	None	NS
Güçer et al. (1988) (66)	Adult: 127	NS	Hydrocephalus, unspecified: 94; IIH: 21; obstructive hydrocephalus: 12	NS	Epidural passive radiotelemetric ICP sensor	Mean (SD): 6.8 (0.44) years	NS	NS	Asymptomatic perisensor epidural hematomas: 2
Maas and de Jong (1986) (67)	Pediatric: 22	NS	Trauma and stress related: 13; hydrocephalus, unspecified: 6; posthemorrhagic hydrocephalus: 2; tumor-related hydrocephalus: 1	Diagnostic evaluation: 22	Rotterdam Teletransducer	NS	NS	1 device removal	Superficial wound infection: 1
Chapman et al. (1984) (69)	Adult and pediatric: 8	NS	Obstructive hydrocephalus: 8	Tumor resection: 8	Custom telemetric monitoring system	NS	NS	NS	None
Nulsen et al. (1980) (68)	Pediatric: 3	Mean (SD): 8.33 (3.79)	Posthemorrhagic hydrocephalus: 1; tumor-related hydrocephalus: 1; obstructive hydrocephalus: 1	Shunt revision or tumor resection	Custom telemetric monitoring system	Mean (SD): 27.3 (11.9) days^k^	39 days	1 pt required shunt revision after telemetry showed high ICP	NS

The etiologies reported in the included studies were classified under the following categories for clarity: obstructive hydrocephalus (including obstructive hydrocephalus, aqueductal stenosis, occlusive hydrocephalus, pineal astrocytoma, tumor, brain tumor, tumor-related hydrocephalus, noncommunicating hydrocephalus, and postaqueductal stenosis); NPH (NPH, idiopathic NPH, secondary NPH); IIH (benign intracranial hypertension, IIH, pseudotumor cerebri); congenital hydrocephalus (congenital hydrocephalus, infantile hydrocephalus, myelomeningocele, meningomyelocele, complex congenital malformation, megalencephaly-capillary malformation syndrome, occipital encephalocele-related hydrocephalus, congenital/complex malformation, complex cerebral malformation, spina bifida); posthemorrhagic hydrocephalus (posthemorrhagic hydrocephalus, intracerebral hematoma, subarachnoid hemorrhage, cerebral hemorrhage); posttumor hydrocephalus (posttumor hydrocephalus, intracerebral gliomas, malresorptive hydrocephalus related to recurrent craniopharyngioma and cerebellar astrocytoma, tumor surgery); Chiari malformation (Chiari malformation, Arnold-Chiari syndrome); craniosynostosis (craniosynostosis, Pfeiffer's syndrome, Crouzon syndrome); postinfectious hydrocephalus (hydrocephalus in infectious diseases, postinfectious hydrocephalus, cerebral infection); shunt-related disorders (shunt malfunction, chronic underdrainage after shunt revision, intermittent shunt dysfunction, slit ventricle syndrome, CSF leak, pseudomeningocele); trauma and stress-related (severe head injury, traumatic brain injury, posttraumatic hydrocephalus, hypoxic brain injury, intracranial hypertension after thrombosis or hemorrhage, brain edema); cystic malformations (Blake's pouch cyst, infratentorial cyst, Dandy Walker variant or syndrome, arachnoid cyst, postarachnoid cyst fenestration hydrocephalus, suprasellar arachnoid cyst, type III arachnoid cyst). CI, confidence interval; CSF, cerebrospinal fluid; ICP, intracranial pressure; IIH, idiopathic intracranial hypertension; IQR, interquartile range; NA, not applicable; NPH, normal pressure hydrocephalus; NS, not specified; pt, patient; SR, sensor reservoir.

^a^ The included children represent a wide section of hydrocephalus diagnoses. Some children have more than 1 diagnosis, and thus the total number of diagnoses does not equal the number of children participating in the study. In addition, the number of devices exceeds the number of patients, indicating that multiple devices were used for some individuals.

^b^ All patients initially recieved Neurovent-P-tel implants; however, because its approved implantation period is limited to 90 days, the device had to be replaced after this time point.

^c^ The first group included patients with suspected CSF disorders, and the second group consisted of those with confirmed CSF disorders.

^d^ Five patients were excluded from this study, 1 of whom had a fulminant shunt infection. The remaining cohort was included in the final analysis.

^e^ Twenty-five patients were included in the study solely for research purposes.

^f^ Although the study initially included 74 patients, only 48 propensity-matched pairs were reported. Additionally, 8 patients were categorized under “other,” which included conditions such as CSF leak, pseudomeningocele, subarachnoid hemorrhage, and aqueductal stenosis. However, as the exact distribution of these diagnoses was not specified, only 40 patients were included in the final analysis.

^g^ One patient had 2 sensors implanted due to infection after receipt of the first implant.

^h^ Nineteen patients underwent implantation of telemetric probes for post-operative monitoring (shunt, ETV, etc.).

^i^Four patients with brain tumors were used as a reference group because they did not have any underlying CSF disorder. Therefore, this group was excluded from the table for the remaining data analyses.

^j^Main indications for ICP measurements were suspected hydrocephalus, exclusion of shunt dysfunction, monitoring after shunt ligature, and patency control after neuroendoscopy.

^k^In descriptive statistics, values indicated as “greater than” (e.g., “>180”) were approximated by the threshold value itself (e.g., 180).

Among the reported timings for telemetric device insertion, the most common was during diagnostic evaluation (*n* = 376), followed by during postoperative surveillance (*n* = 329), at the time of primary shunt or ETV surgery (*n* = 264), and at the time of shunt or ETV revision surgery (*n* = 186). Insertion during tumor resection was reported in 10 cases, and the timing was not specified in 246 cases (Figure 3).

The most frequently reported complications were neurological events (*n* = 21) and skin-related issues, including irritation and erosion (*n* = 22). These were followed by shunt-related complications (*n* = 11), wound infections or dehiscence (*n* = 10), and discomfort or cosmetic concerns (*n* = 9). Pain was reported in 7 cases, and nonshunt, non-wound-related infections were documented in 5 cases. Hemorrhage or hematoma occurred in 6 cases (Figure 3 and Table 2).

The functional duration of telemetric devices was reported, along with statistical details, in 14 studies. Based on these, the pooled average functional duration was calculated to be 529.41 (836.94) days (Table 2). However, because the studies did not consistently associate specific device types with their respective durations, direct comparison of functional lifespan between different telemetric systems was not feasible.

## 4 Discussion

ICP can increase due to various neurosurgical pathologies, including hemorrhages, tumors, infections, and both primary and secondary hydrocephalus (72–78). Therefore, continuous ICP monitoring plays a critical role in guiding timely intervention, especially in hydrocephalus management (54, 79). Hydrocephalus treatment primarily focuses on correcting underlying CSF circulation disturbances through surgical diversion methods such as cerebral shunts and ETV. However, surgical intervention alone is not always sufficient; effective long-term management is also essential. This ongoing care relies on ICP monitoring to guide diagnosis and treatment, particularly in complex cases involving shunt systems, to optimize patient outcomes (16, 25, 37, 48, 49). Conditions such as NPH, congenital hydrocephalus, idiopathic intracranial hypertension, postcraniotomy communicating hydrocephalus, and posthemorrhagic hydrocephalus often present with complex and fluctuating symptoms, requiring precise and continuous ICP measurement (27, 30, 32).

Although technological advancements have improved CSF drainage techniques, determining optimal valve adjustments remains largely dependent on clinical and radiological findings. These methods may not always be sufficient to accurately detect abnormal ICP changes (9). Telemetric sensors have emerged as a potential solution, offering continuous, noninvasive ICP monitoring for postoperative surveillance, particularly for shunt-treated patients (16, 38). Since their initial development, 8 different models of telemetric ICP monitoring devices have been produced and clinically tested to date.

### 4.1 Clinically tested telemetric devices and their operating principles

#### 4.1.1 Radionics TeleSensor (1978)

**Figure 4:**
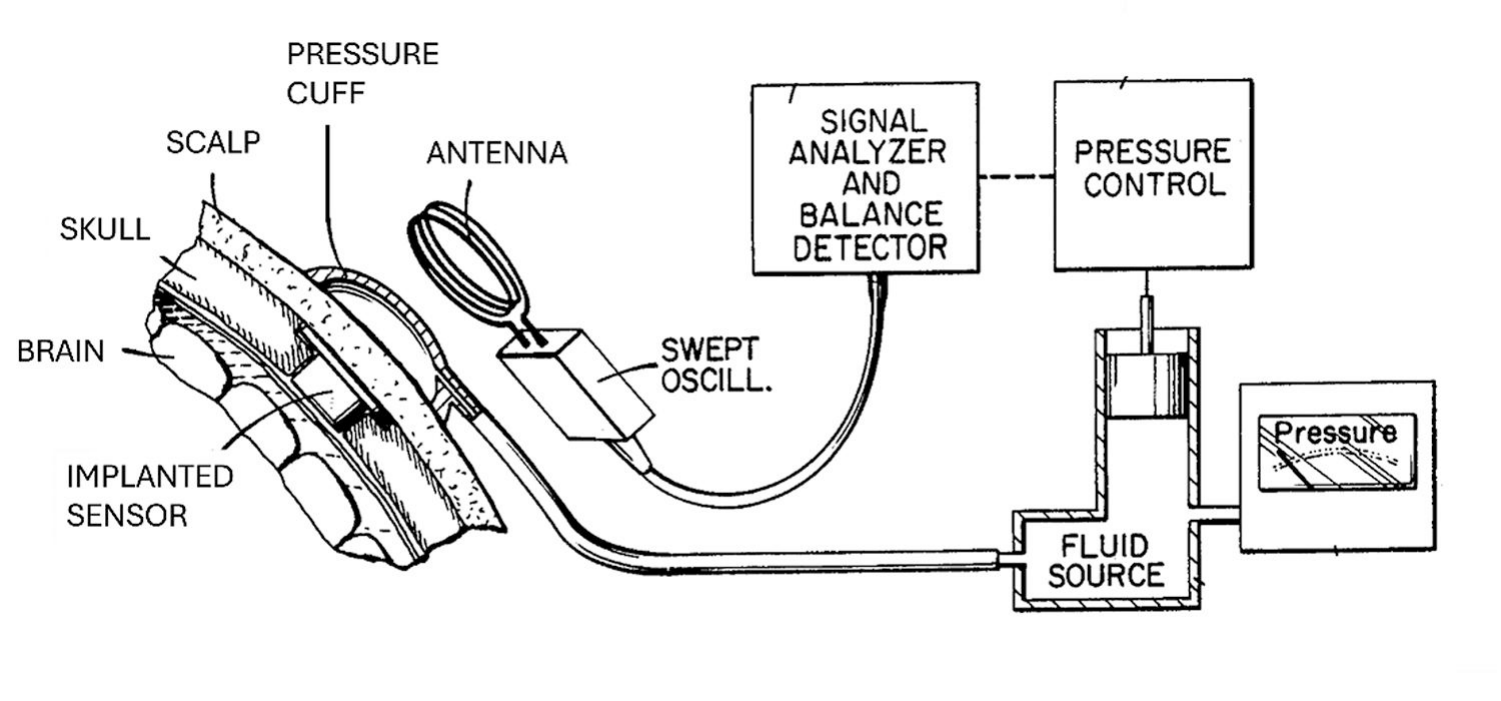
Diagram of Radionics TeleSensor implant and readout system, including pressure source used to counterbalance the intracranial pressure at the sensor. The diaphragm is built with a stop, which prevents movement of the inductor core past a fixed position. The diaphragm can expand freely but cannot compress to less than its rest position (the position at zero difference between intracranial and subgaleal pressure). The stop serves as a reference point, which is essential for the calibration and readout of the sensor (107). Figure is in the public domain. Retrieved from https://patents.google.com/patent/US4653508A/en.

First described in 1972 and later produced by Radionics, Inc. (Burlington, MA, USA), this sensor uses an inductor and capacitor to form a resonant circuit with passive readout (80). The inductor consists of a coil of wire wrapped around a ferrite core mounted on a movable diaphragm. CSF pressure pushes the diaphragm, moving the ferrite core and thereby changing the resonant frequency. As shown in Figure 4, the resonant frequency is measured by an external antenna using the grid dip method: the external reader applies a frequency sweep to find the resonance peak.

A pressurized air cuff is required for measuring ICP using a pressure-balancing method. For each reading, the sensor is first calibrated by manually pushing the diaphragm against its internal stop and adjusting the readout meter to the calibration line. This line marks the resonant frequency with the diaphragm in its resting position. When the manual pressure is released, internal pressure deforms the diaphragm, which shifts the resonant frequency. To measure ICP, the air cuff is inflated against the scalp, applying increasing pressure until the resonant frequency reaches the calibration mark. At this point, the internal and external pressures are balanced, so the air cuff pressure equals the ICP.

Clinical tests in patients with hydrocephalus showed the utility of this device, with sensors implanted for 6 months experiencing no drift and no loss of sensitivity (80). The absence of drift can be attributed to the recalibration of the sensor during each use. The accuracy is within 10–15 mm H_2_O, although this is only when the scalp is not edematous; edema introduces an unknown offset in the pressure reading. The sensor proved useful for managing obstructive hydrocephalus after tumor treatment (69). Cardiac pulsation in the ICP waveform indicated the patency of the ventricular catheter: when the inlet was occluded, the cardiac waveform was dampened.

A vacuum source is required for reading negative pressure (65). One study measured the ICP vs. posture curves with different valves (61). Flow-limited and antisiphon valves showed ICP vs. posture curves similar to those for nonhydrocephalic patients. In patients without shunt placement, the natural antisiphon behavior is mediated by neck veins collapsing when upright, acting as flow-limiting “Starling resistors.”

The sensor has been tested with adjustable valves, showing that ICP depends not only on valve settings but also on the absorptive site (peritoneum, atrium, or pleura), other shunt components (e.g., antisiphon device), and posture (supine vs. head elevated) (56). Ventriculopleural shunts yielded lower ICP because of negative pleural pressure (60).

This telesensor can monitor ICP during recovery from an ETV for noncommunicating hydrocephalus (61). Flatter ICP vs. posture curves after the ETV showed the recovery of brain compliance. Of note, the same investigator performed all the measurements, which was necessary to address the interoperator variability inherent in the pressure balance method (58).

Advantages of this system include that it uses an entirely passive implant with no battery and has a fast dynamic response that shows cardiac pulses. Disadvantages include a cumbersome pressure balance method requiring an external pressure cuff and consistent operator (58) and the need for a vacuum source to measure negative pressure (65).

#### 4.1.2 Case Western Reserve University sensor (1980)

Developed by engineers and neurosurgeons at Case Western Reserve University (CWRU) (Cleveland, OH, USA), this design includes several features common to more modern sensors. Unlike the Radionics sensor, which measures ICP relative to an externally applied pressure, the CWRU device uses a sealed case to hold an internal vacuum reference, thereby measuring the absolute pressure. The pressure signal from the silicon piezoresistor is amplified by onboard active electronics (e.g., transistors) and sent to the external reader by an onboard transmitter. Unlike most other designs, the CWRU device is battery-powered.

Clinical tests demonstrated the usefulness of the CWRU device for managing hydrocephalus over time (68). Initial tests uncovered issues addressed in the second-generation design: (1) device performance was variable, (2) gold foil isolating the sensor was easily damaged, (3) packaging degraded after several months, and (4) the individual circuit components were challenging to assemble reliably (81, 82).

**Figure 5:**
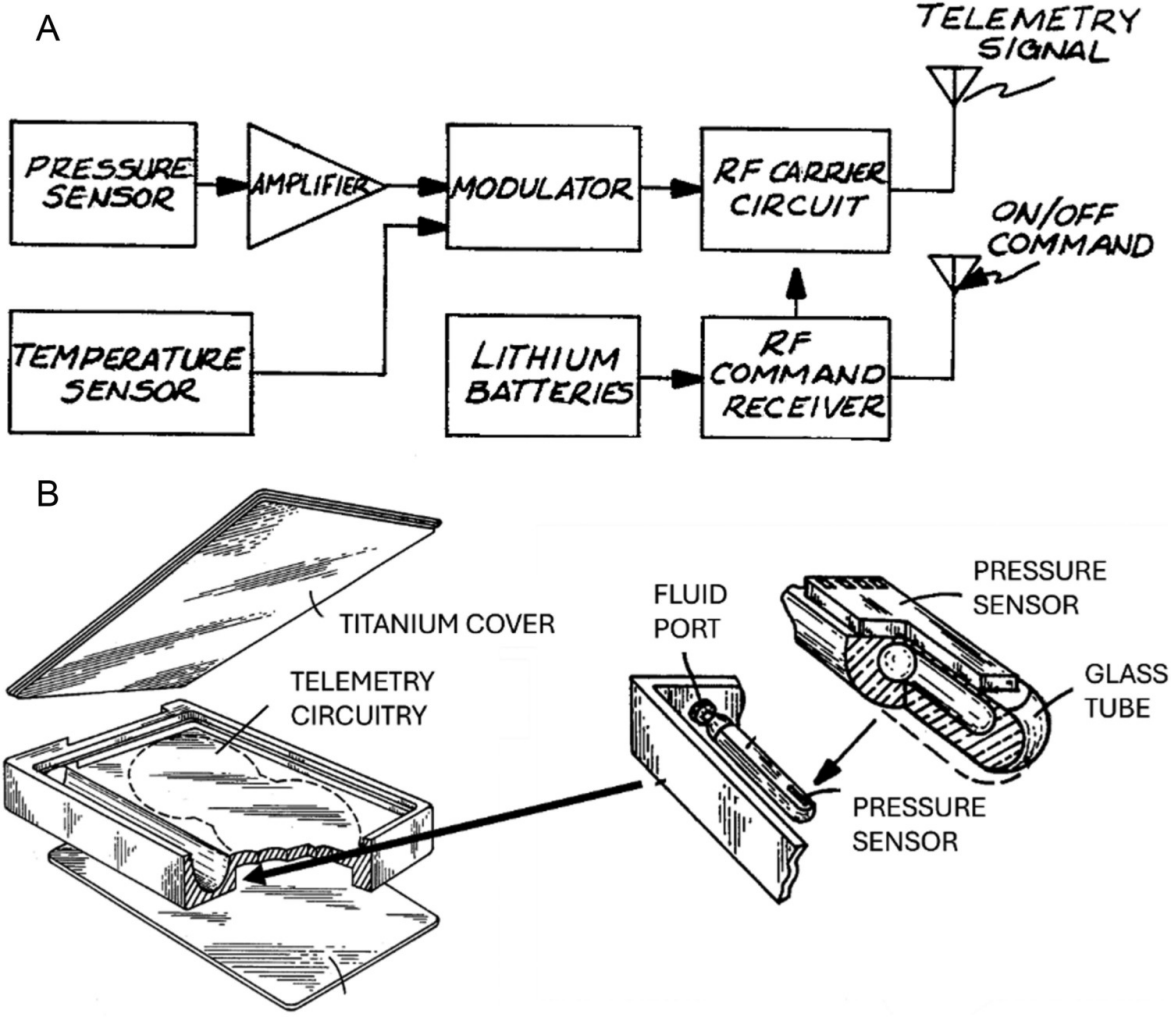
A system architecture diagram and a schematic of the entire device illustrating the Case Western Reserve University (CWRU) telemetry system (108). **(A)** Block diagram of the CWRU telemetry system. The pressure sensor resistance reading is amplified and temperature-compensated, then the data are transmitted as a radiofrequency signal to the external receiver. The external receiver sends an on/off signal to activate the sensor. **(B)** Illustration of the CWRU sensor components. Left: Full case, showing housing for the readout circuitry and the circular slot for the pressure sensor. The case (29 mm × 20 mm × 7 mm) is hermetically sealed, and the vacuum inside is the absolute reference pressure for the sensor. Middle: A closer view of the pressure sensor, with the Pyrex glass tube sealed to the titanium adapter connecting to the ventricular catheter. Right: A closer view of the silicon piezoresistive pressure-sensing diaphragm fused over a small opening in the glass tube. Figure is in the public domain. Retrieved from https://patents.google.com/patent/US4519401A/en.

Packaging failures arose from moisture saturating the encapsulant, which can cause electrical leakage between wires and deformation of the pressure transducer. This issue was resolved using a laser-welded titanium case. Consistency was improved by simplifying the circuit, and battery life was enhanced by using a radiofrequency-controlled power switch to activate the system when needed (81, 82). A silicon piezoresistor was used as the pressure-sensing element; although it was later proposed that a capacitive pressure sensor be used instead (83). A diagram of the system architecture, including details of the glass-mounted silicon pressure sensor, is shown in Figure 5. Notably, the CWRU sensor was added to the shunt with a 3-way “T” connection, with the sensor as a blind end without an outlet.

#### 4.1.3 Osaka telesensor (1981)

**Figure 6:**
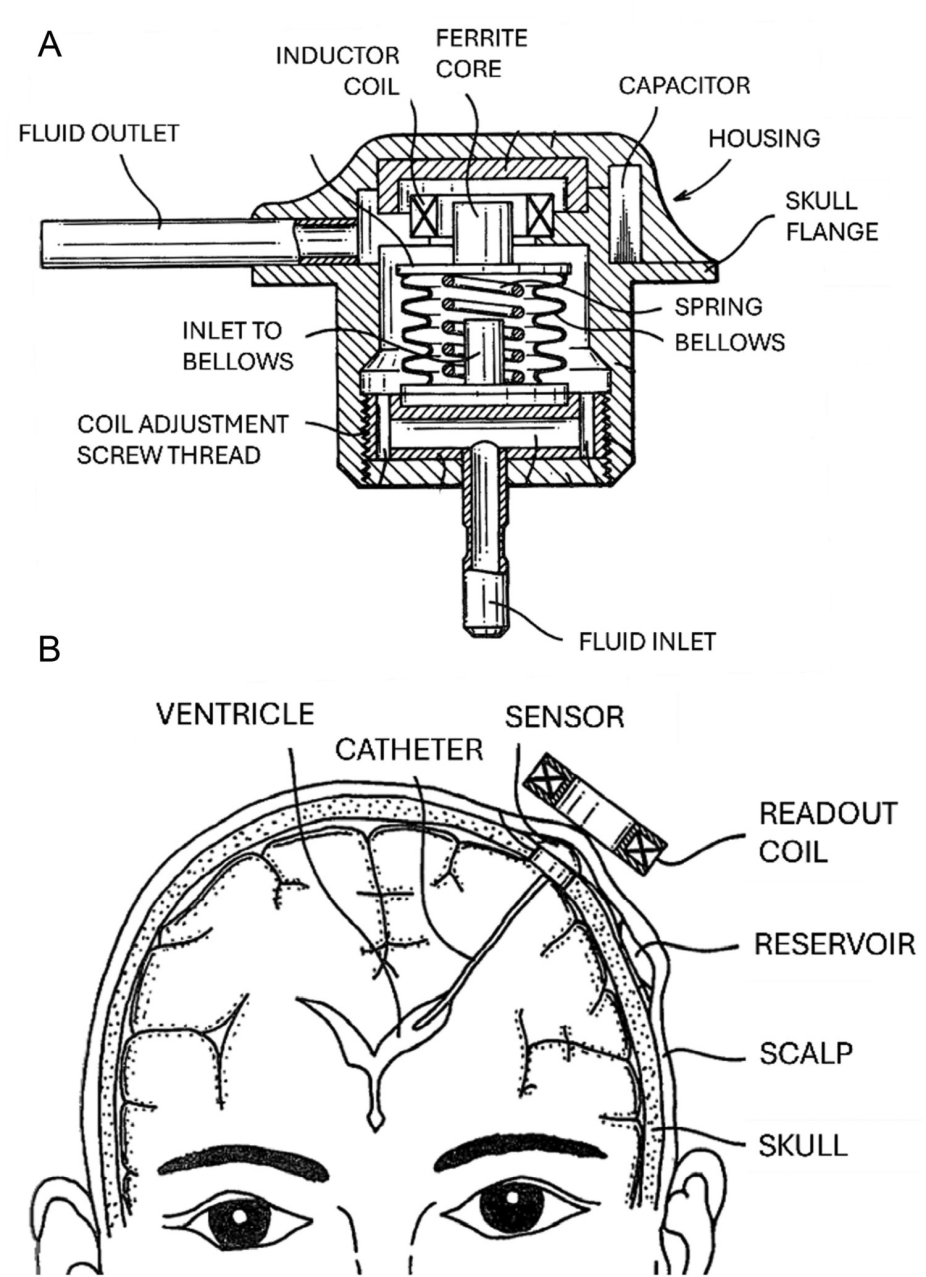
The Osaka telesensor (109). **(A)** A cross-sectional schematic of the Osaka telesensor. The inlet receives cerebrospinal fluid (CSF) from the ventricular catheter. The fluid pressure pushes against the spring to expand the bellows, with the ferrite core attached to the free end. The relative position of the ferrite core inside the coil determines the inductance. This pressure-varying inductance and the fixed capacitance set the resonant frequency. The coil also serves as the antenna for coupling to the external reader device. **(B)** A diagram of the sensor as implanted with a ventricular catheter and CSF reservoir, with the readout coil placed against the scalp for measurement. Figure is in the public domain. Retrieved from https://patents.google.com/patent/US4354506A/en.

The Osaka telesensor (Nagano Keiki Seisakusyo Co Ltd, Tokyo, Japan) was described in 1981 (63, 84). It is an entirely passive device with no batteries, transistors, or other active electronics (Figure 6). Like the Radionics design, pressure is sensed by the movement of a ferrite coil within an inductor coil. This inductor forms part of a resonant circuit. Unlike the Radionics device, the Osaka telesensor has a vacuum-sealed case with an internal reference. The handheld reader unit includes a barometer to subtract this ambient pressure from the signal. The sensor is coupled to an external antenna that detects resonance using the grid dip method for readout. Once the resonant frequency is identified, the reader converts this into absolute pressure based on earlier benchtop calibration (84).

In clinical testing including patients with hydrocephalus, the Osaka telesensor proved helpful for postoperative care (e.g., diagnosing shunt failure and adjusting valves). The pulse waveform and the ICP response to reservoir pumping provided supplementary information: pulse waves are absent in cases of ventricular catheter occlusion or slit ventricle syndrome (71). The ICP response to postural changes offered insights into cerebral compliance. The Osaka telesensor exhibited significant zero drift over time, as measured by puncturing the shunt reservoir (71).

#### 4.1.4 Rotterdam Teletransducer (1984)

The RTT (Erasmus University, Rotterdam, Netherlands) uses a passive design similar to the Radionics and Osaka sensors but with a pressure sensor based on changing capacitance rather than changing inductance. The handheld interrogation system is similar to the Osaka, using an external coil to sense the implant's resonant frequency and an onboard barometer to convert from absolute pressure to ICP.

The RTT is recognized as a noninvasive fontanelle pressure sensor for infants, but it has also been tested as an epidural sensor (59, 62, 85). The RTT is a passive device using an inductor-capacitor resonant circuit. The sensing element is the capacitor, which is made of a pressure-deformable titanium diaphragm positioned parallel to a silver plate at a 50-µm distance. The interior of the sensor must remain vacuum-sealed to maintain the zero reference pressure. Titanium was selected for its inertness and strong bonding to the ceramic housing. The ceramic encapsulation with glass or metal brazing joints facilitates radiofrequency transmission and minimizes moisture and gas penetration. Earlier prototypes with epoxy resin encapsulation exhibited tolerable drift for up to 2 months of use only (67).

**Figure 7:**
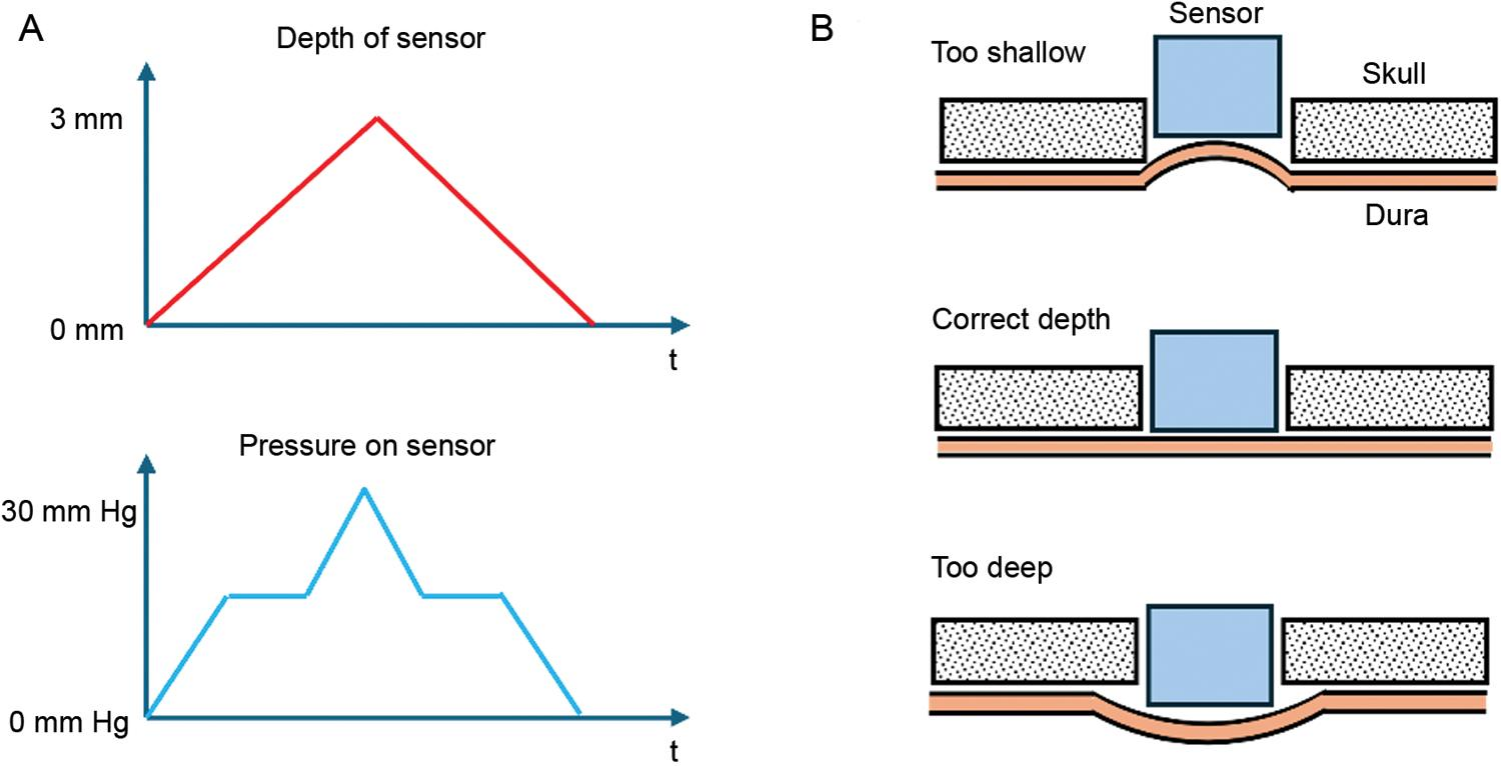
Pressure-depth curve diagrams used during Rotterdam Teletransducer placement. **(A)** Diagram of the pressure-depth curve used during Rotterdam Teletransducer placement to verify correct insertion depth for valid pressure sensing. This diagram is based on data from fontanelle pressure measurements, but the procedure for epidural sensing is similar. A calibration screw is used to advance the transducer into closer contact with the dura while recording the pressure reading. The starting position (d = 0 mm) only lightly touched the fontanelle and showed near zero pressure. As the depth was increased from 0 up to 3 mm, the pressure first increased, then plateaued, then increased sharply again. The plateau region, where the pressure is constant vs. depth, corresponds to the appropriate contact of the transducer on the fontanelle. The sharp increase after the plateau shows overinsertion when the sensor begins to deform the fontanelle or dura. Note that, in real data, the cardiac and other pressure waves are superimposed on the pressure-depth curve (67). **(B)** Diagram of the pressure-depth principle. When the transducer is inserted too shallowly, the sensor does not experience the full pressure from the epidural surface because some of the pressure is supported by the skull instead. When inserted correctly, the pressure is uniformly distributed across the skull and the sensor. When inserted too deeply, the sensor begins pushing the dura away from the skull, and therefore, the sensor experiences pressure from an area wider than the sensor face itself. Used with permission from Barrow Neurological Institute, Phoenix, Arizona.

The cylindrical assembly was designed to fit into a standard burr hole craniotomy, with a diameter of 10 mm and a height of 7 mm. Mounting hardware secures the RTT, with a large screw attaching the steel clip to the skull and a small calibration screw used for fine adjustment of the depth. The depth must be carefully adjusted for accurate pressure readings. The transducer was placed by eye in early clinical tests, resulting in errors. A pressure-depth curve was used in subsequent tests to set the transducer position (Figure 7). The curve is created by measuring the apparent pressure as the transducer position is swept over a range of depths. This calibration method could be helpful for any skull-mounted epidural pressure sensor.

#### 4.1.5 Johns Hopkins sensor (1988)

Developed by Johns Hopkins University (Baltimore, MD, USA), this sensor shares many design elements with the RTT. It is a passive device with a pressure-sensitive capacitor and a coil antenna, together forming a resonant circuit with a frequency near 50 MHz for readout by an external coil (66). The sensing capacitor has 2 plates, one fixed in place and the other mounted on expandable bellows. The polycarbonate outer enclosure is filled with silicone fluid, which transmits pressure from the outer diaphragm to compress the bellow. Unlike other sensors designed for direct CSF contact, the Johns Hopkins sensor enclosure is designed for epidural placement through a burr hole craniotomy, with the outer diaphragm placed against the dural surface.

Across an average clinical test duration of 6 years, the sensor showed a significant drift of up to 1 mm H_2_O per day (66). Drift was measured by comparing the sensor data with lumbar puncture readings. Several mechanisms caused drift: (1) The plastic enclosure is gas permeable, and the silicone fluid has a high affinity for absorbing gas, thereby creating internal pressure. Some explanted devices were visibly bulging. (2) The nickel bellows of the capacitive sensor had some porosity, which was observable in a helium leak test, and this caused nitrogen gas loss over time. (3) Explanted sensors had fatigue cracks in the plastic and discoloration of internal parts, showing corrosion by fluid ingress.

#### 4.1.6 ICP-Telesensor (1999)

The ICP-Telesensor was built in collaboration between Heinrich-Heine University (Düsseldorf, Germany) and Telemeasurement GmbH (Würselen, Germany) (58). Like the Johns Hopkins sensor discussed above, the ICP-Telesensor involves a passive design that uses a pressure-deformable capacitor. The capacitor shifts the implant's resonant frequency, which is interrogated by an external readout coil.

A 0.1-mm titanium membrane serves as the deforming capacitor membrane. The sensor is a flow-through device and is placed between the ventricular catheter and the valve. As a sealed absolute pressure sensor, the reader must compensate for barometric pressure to find the ICP. The sensor was safe and useful for hydrocephalus management in preliminary clinical testing (58). The capability to measure pressure across various head elevations provides valuable information, and the readout method is simple enough to be performed by nurses or patient family members.

#### 4.1.7 Neurovent-P-tel (2009)

Unlike the sensors described above, the final 2 sensors discussed herein, the Neurovent-P-tel and the Miethke Sensor Reservoir (discussed below), have recently been used in clinical settings. Improvements in sensor reliability have enabled their wide adoption. These 2 designs use active electronics to amplify the sensor signal, but unlike the CWRU sensor, they are wirelessly powered. The amplification allows for a more accurate readout and more efficient communication with the external side.

**Figure 8:**
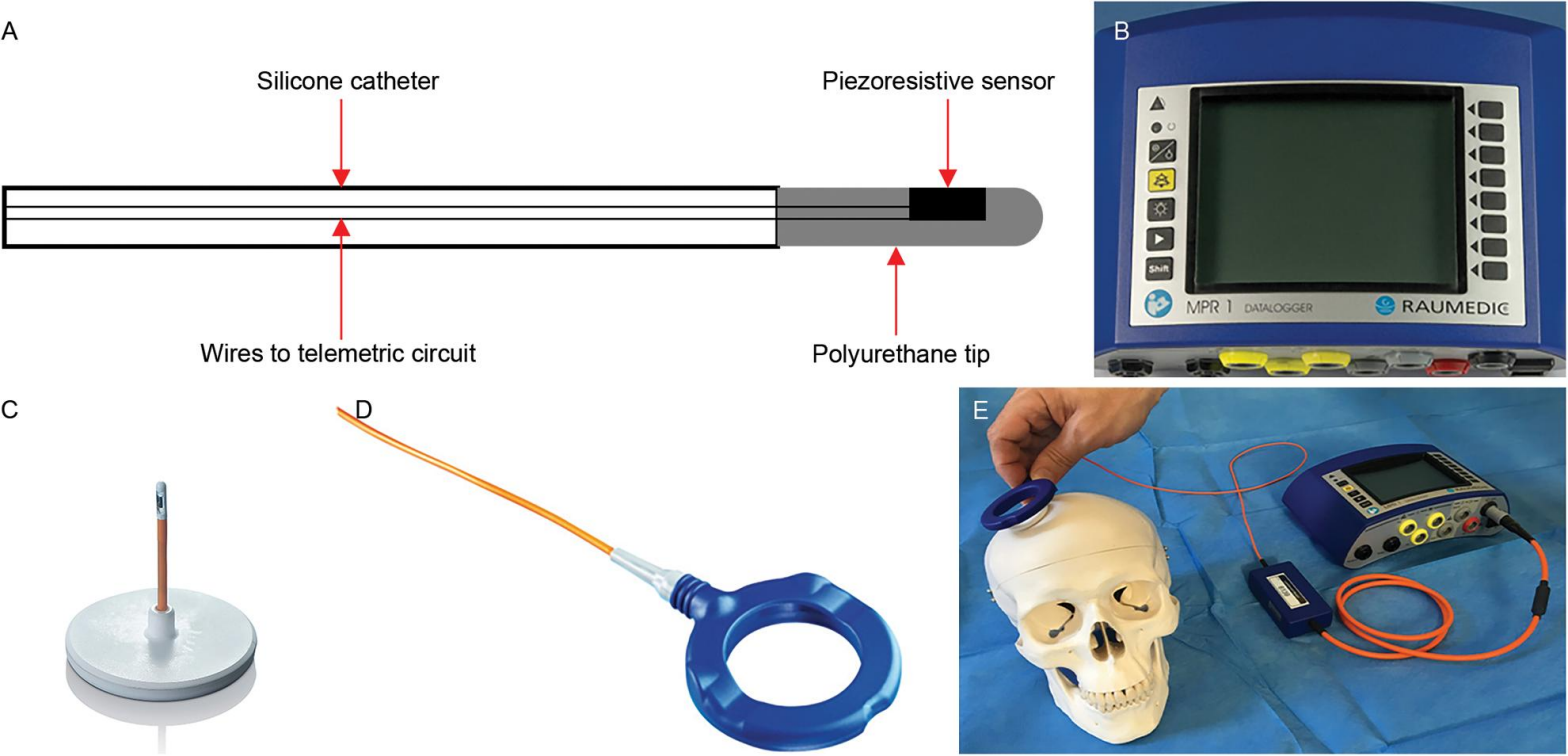
Raumedic Neurovent-P-tel telemetric intracranial pressure (ICP) measurement device (86). **(A)** Diagram of the sensing catheter used in the Raumedic Neurovent-P-tel. **(B)** The DATALOGGER, also referred to as an interactive display and storage unit. **(C)** Implantable P-tel unit. **(D)** The antenna (reader) unit. **(E)** The Neurovent ICP monitoring system and its placement during an ICP monitoring session. The P-tel catheter is typically implanted in the right or left frontal lobe beneath the scalp, whereas the circular reader is positioned on the skin directly above the catheter and secured with adhesive tape to enable measurement. Figure is in the public domain. Retrieved from doi: 10.1186/s12883-021-02349-8.

The Neurovent-P-tel was launched by Raumedic (Helmbrechts, Germany) in 2009 (16). Unlike most other sensors connecting to the CSF shunt system, this freestanding sensor includes a parenchymal catheter. Near the tip of the polyurethane catheter, the piezoresistive pressure sensor is mounted on a flexible membrane. Pressure deforms the membrane, altering the length of the piezoresistors and changing the resistance measurement (16, 48, 51). The 25-mm catheter is inserted into the parenchyma through a burr hole. As shown in Figure 8, the ceramic disc base rests on the skull surface. When used alongside a shunt, the Neurovent-P-tel is implanted on the opposite hemisphere from the ventricular catheter (16, 32).

The telemetry unit is housed in ceramic, allowing effective data transmission. Ceramic is impermeable to water vapor, protecting the circuits from corrosion. The ceramic base connects to the silicone-coated polyurethane parenchymal catheter, with the pressure transducer at its distal end (51). The sensor has a sampling rate of 5 Hz, which is lower than that of conventional ICP sensors. This lower sampling rate reduces the resolution of pulse waves for waveform analysis but is sufficient for measuring average ICP (35). The telemetry system employs a coil antenna and a microchip housed within a ceramic casing. The implanted antenna interacts with the electromagnetic field generated by the external handheld reader unit, and the microchip transmits data by applying load modulation to the implanted antenna. The external reader unit captures the resulting variations on the external antenna (16). The reader then displays and stores the collected data (Figure 8) (86).

Preclinical testing demonstrated high accuracy, with zero-point drift of 2.5 mm Hg over 18 months (31, 87). In human studies, zero drift was 4–5 mm Hg over 11 months (87). According to manufacturer specifications, the device should not be implanted for more than 90 days. Postapproval monitoring found that the Neurovent-P-tel had a 3.1% chance of packaging failure when used for longer recordings. The packaging failures caused drift due to moisture absorption in the analog circuitry. Although there are no reports of patients having been harmed, Raumedic has withdrawn the sensor from long-term use (88). One of the other reasons behind this withdrawal was early sensor malfunction, more specifically, premature signal failure shortly after implantation, which compromised Neurovent-P-tel's reliability for long-term monitoring (88).

The Neurovent-P-tel sensor offers several advantages for both in-clinic and at-home care (89, 90). The sensor is usually reliable for long-term ICP measurement, with minimal zero drift and strong correlation in waveform curve analysis (87). These characteristics make the sensor well-suited for ICP monitoring during the 8- to 12-week recovery period following ETV. The sensor can measure ICP while the patient is supine or standing, aiding in identifying posture-related over- and under-drainage.

The low sampling frequency of 5 Hz poses a disadvantage. This limits pressure curve analysis, although measuring pulse pressure amplitude remains feasible (90). Baseline drift can compromise accuracy: studies indicate a median shift of 2.5 mm Hg from baseline, and the amount of drift is especially large when implant duration exceeds the Conformité Européenne (CE) mark limit of 90 days (37). This risk is among the factors that led to the device being withdrawn from the market for long-term use (88). As with all implants, complications can occur: a study of 247 patients found a 1.6% risk of superficial wound infection, a 0.8% risk of brain abscess, and a 0.4% incidence of clinically significant intracerebral hemorrhage, although all complications typically resolved within 2 weeks (35). The sensor can also become encapsulated by a biological coating on its tip, forming a sleeve that encases the sensor; fortunately, documented instances in clinical practice are rare (37).

#### 4.1.8 Miethke Sensor Reservoir (2015)

The Miethke Sensor Reservoir (now marketed under the brand name M.Scio) was introduced in 2015 (38). Similar to several earlier designs (Radionics TeleSensor, CWRU sensor, Osaka telesensor, Johns Hopkins sensor, and the ICP-Telesensor), this modern sensor is a flow-through device fitting into existing shunt systems. Unlike those earlier designs, the Miethke Sensor Reservoir also replicates the functions of a conventional reservoir: it holds a volume of CSF, the silicone membrane on top can be punctured to withdraw CSF or administer drugs, and the reservoir can be palpated to sense outflow resistance and refilling rate for shunt failure diagnosis. As with the Neurovent-P-tel, the improvements in encapsulation and wirelessly powered sensing and communications electronics have enabled accurate long-term performance.

The Miethke Sensor Reservoir features 2 ports: the inlet connects to the ventricular catheter, and the outlet leads to the valve. The sensing unit is housed in a 12-µm-thick titanium casing within the reservoir. The Miethke Sensor Reservoir uses 64 capacitive pressure sensors. Pressure deforms the flexible diaphragm, altering the gap between the capacitor plates. The resulting change in capacitance is measured by onboard circuitry and correlated back to pressure based on calibration for the sensor size and materials (91).

The Miethke Sensor Reservoir is a passive battery-free implant that relies on the external handheld reader unit to provide wireless power through magnetic coupling. The handheld reader includes the coil antenna, a display, and storage. A sampling rate of 44 Hz allows for the accurate capture of the ICP waveform (24).

As with all telemetry systems, the advantage of noninvasive sensing is tempered by concerns about accuracy. Prolonged mechanical stress and aging of electronic components can lead to measurement drift. The sensor is rated for implantation for 3 years by CE, having been tested for 2.5 years with a measurement accuracy of 1 mm Hg (35). The system is also unsuited for continuous pressure monitoring because the sensor is passive and requires continuous power by maintaining the reading unit on the correct scalp location.

### 4.2 Clinical implications

The renewed popularity of telemetric ICP monitoring devices began approximately 16 years ago with the licensing of the Neurovent-P-tel (90). The clinical use of these devices gained more popularity in 2015 with the introduction of Miethke's SR. However, despite their increasing adoption, there are currently no standardized guidelines governing their clinical implementation.

Telemetric ICP monitoring devices have shown applicability in various neurosurgical conditions requiring ICP assessment. They have been used in acute settings such as traumatic brain injury within the intensive care unit, with data quality and long-term stability considered sufficient for clinical decision-making based on average ICP values. In addition to their utility in treating acute pathologies, these devices are valuable in the long-term management of hydrocephalus and other conditions such as craniosynostosis and pseudotumor cerebri (54, 92).

These devices can be used for diagnostic purposes and to monitor treatment responses. A study by Riedel et al. in which patients with hydrocephalus and coexisting sleep apnea underwent simultaneous ICP recordings and polysomnography provides an example of the diagnostic use of these devices (93). That study identified that nocturnal transient ICP elevations were associated with sleep apnea and were reduced after continuous positive airway pressure therapy (93).

Beyond the diagnostic insights that they can provide, these devices are also valuable in predicting treatment outcomes, particularly following ETV. Their ability to provide long-term ICP measurements is especially helpful between postoperative weeks 8 and 12, when ETV failures are most commonly reported (48, 90). As reported by Antes et al., the Neurovent-P-tel implantation during neuroendoscopic ETV in 25 patients with occlusive hydrocephalus was crucial for distinguishing ETV responders from nonresponders. Six of these patients were identified as nonresponders based on an increase in mean ICP during follow-up. The same study emphasized that continuous ICP monitoring is the safest method for detecting conditions such as shunt malfunction and ETV nonresponsiveness (16, 21). Furthermore, a key clinical application, central to the focus of our study, is their use in monitoring shunt effectiveness and verifying sustained shunt function over time in patients with complex hydrocephalus (54).

There are some differences in the clinical applications of the 2 most recent sensors used in telemetric ICP monitoring: the Neurovent-P-tel and the Miethke Sensor Reservoir (Table 3). Although the Neurovent-P-tel has an implantation limit of 90 days, the Miethke Sensor Reservoir does not have a strict technical time limitation; however, its CE certification currently permits implantation for up to 3 years (90). Furthermore, although the Neurovent-P-tel is implanted intraparenchymally and used primarily for diagnostic ICP monitoring, the Miethke Sensor Reservoir must be integrated into a shunt system, and its primary use has been reported as ICP-guided adjustment of shunt valves in adult and pediatric patients with hydrocephalus (Table 3) (38, 90).

**Table 3:** Comparison of key technical features of the Neurovent-P-tel and Miethke Sensor Reservoir systems.

Feature	Raumedic Neurovent-P-tel	Miethke Sensor Reservoir
Implantation duration	90 days	3 years (CE approval)
Surgical implantation	Placed through a frontal burr hole	Integrated into a shunt system
Placement	Parenchymal	Ventricular
Need for additional removal surgery	Yes	No
Sampling frequency	1–5 Hz	40–44 Hz
Primary clinical application	Diagnostic purposes	Continuous and repeated ambulatory measurements
Monitoring the success of ETV procedures	Can detect drainage-related shunt dysfunction	Can guide shunt valve setting adjustments
MRI compatibility	Yes, for 1.5 and 3 Tesla	Yes, for 1.5 and 3 Tesla (recalibration may be required)
Associated software	RAUMED DataView	ICPicture
Implant longevity and monitoring utility	Extended monitoring periods	Extended implant duration allowing for multiple brief ICP assessments
Clinical use	Withdrawn from the market	Actively in use

ETV, endoscopic third ventriculostomy.

When considering the primary clinical indications for each device, Neurovent-P-tel is best suited for diagnostic purposes or for monitoring the success of ETV procedures. In contrast, the Miethke Sensor Reservoir is more appropriate for continuous or repeated ambulatory measurements, making it ideal for long-term outpatient follow-up. Its clinical value lies in detecting subtle, drainage-related shunt dysfunction and in guiding valve setting adjustments during serial evaluations over extended periods (90).

The use of the Neurovent-P-tel is relatively simple. Most physicians or nursing staff can be trained within minutes to operate the system. With brief instructions on configuring the display and storage monitor, securing the antenna to the scalp with adhesive tapes or bandages, and supervising patients during monitoring, healthcare providers can easily manage the process (16). Due to the system's simplicity, many patients can also manage the procedure independently at home. This practicality enables ICP monitoring under everyday conditions (16). The ICP values measured by the Neurovent-P-tel system can be analyzed using the accompanying software package, RAUMED DataView, which provides basic analysis of ICP measurements, thereby facilitating clinical interpretation (35). In contrast, the Miethke Sensor Reservoir system is supported by ICPicture software, which enables comprehensive visualization, analysis, and documentation of ICP data (25).

Despite their clinical utility, these sensors also present certain practical challenges. For instance, the Miethke Sensor Reservoir system utilizes a large radio frequency identification antenna as a part of its reading device, which must be positioned directly over the reservoir to measure and store ICP values (31, 35). Permanent fixation of this relatively heavy antenna on the patient's head is not feasible, thereby limiting the ability to perform extended ICP monitoring (e.g., overnight or 24–48-hour recordings) (31). Furthermore, due to its height (approximately 7.7 mm), undesirable cosmetic outcomes can occur following implantation. A visibly noticeable swelling can develop, and over time, there is a risk of wound dehiscence over the Miethke Sensor Reservoir site (31).

For Raumedic probes, secure fixation, such as suturing or taping, is necessary to maintain a stable connection during prolonged measurements. If the connection is disrupted, the datalogger is programmed to emit an audible alarm, alerting healthcare providers. Although this feature enhances safety, it can disturb patients and interfere with uninterrupted data acquisition. Therefore, meticulous attention to maintaining secure connections is essential to ensure reliable signal transmission and data continuity (16).

### 4.3 Insertion and removal of the most recent telemetric ICP monitoring devices

The surgical implantation of the Neurovent-P-tel is a relatively simple procedure that can be performed under either general or local anesthesia (16). After a short linear skin incision of approximately 4 cm is made, a precoronal parasagittal burr hole is created, through which the catheter is advanced into the frontal brain parenchyma (16). The final position is reached when the round ceramic housing is seated on the skull surface. It is recommended to close the incision with sutures rather than staples, because metal staples can interfere with the transmission of telemetric ICP data (16). The entire implantation procedure typically takes around 20 minutes.

Because the Neurovent-P-tel is approved for use for up to 90 days, device removal is required after approximately 3 months (16). This is also a minor procedure and can be performed under local anesthesia. The previous incision is reopened, and the ceramic housing is exposed. Once the housing is elevated, the probe is carefully withdrawn. To reduce the risk of CSF fistula formation, placement of a gelatin sponge at the removal site is advised. The removal procedure is generally reported to take no longer than 10 minutes (16).

Because the Miethke Sensor Reservoir is integrated into shunt systems and implanted and explanted together with them, they do not require an additional removal surgery (35).

### 4.4 Use of the telemetric systems in clinical settings

Use of telemetric systems in clinical settings has been limited, and thus analysis of use does not come from large studies. Most data originate from the Miethke Sensor Reservoir and Neurovent-P-tel systems. Although the Neurovent-P-tel has been withdrawn from the market, the Miethke Sensor Reservoir remains in clinical use.

For both systems, initiating an ICP recording session requires placing the external reader unit over the implanted passive sensor. Once the transducers are activated with radio frequency identification technology, ICP waveforms become immediately visible (35). For the Neurovent-P-tel, securing the reader unit with a bandage helps maintain stable and uninterrupted monitoring sessions, especially for long-term use. The system can typically store up to 3 days of data before reaching full storage capacity. In contrast, establishing long-term recordings with the Miethke Sensor Reservoir system can be more challenging, because the reader unit can easily lose connection with the implant. To obtain reliable readings, the reader must be held at a specific distance from the implant (35). However, short-term positional measurements are feasible by manually holding the reader at the appropriate angle and proximity. The Miethke Sensor Reservoir system can store 40 hours of data (35).

### 4.5 Advantages and disadvantages of telemetric ICP monitoring systems

Numerous studies have reported the benefits of ICP-guided adjustments in optimizing shunt settings and improving long-term patient management (16, 24, 25, 32, 33, 37, 38, 50). Conventional ICP measurement methods, such as external ventricular drains (EVDs) or wired ICP probes, are invasive, pose a risk of infection, and are limited to short-term use (typically around 10 days for conventional probes). Additionally, they require hospital-based settings due to their dependence on intensive care unit infrastructure. Unlike conventional methods, which can require multiple interventions, telemetric sensors provide a minimally invasive alternative. They enable long-term monitoring beyond the hospital without restricting patient mobility. These methods stand out for their ability to monitor ICP in both hospital and home settings (21, 51). By wirelessly transmitting data collected through various techniques, they facilitate long-term ICP monitoring even during daily activities and different postures (e.g., lying, sitting, standing) (25, 41, 50).

A recent systematic review by Omidbeigi et al. reported the complication rate associated with telemetric ICP monitoring devices to be 7.1% (94). In comparison, EVD and conventional intraparenchymal ICP monitoring were associated with complication rates of 14.5% and 10%, respectively (95, 96). Among studies involving telemetric ICP devices, the most frequently observed complication was a single new-onset seizure, occurring in 3.9% of cases (51). Reported infection rates for EVDs range from 7.3% to 10.4%, with a notable increase linked to prolonged monitoring duration (97–100). Hemorrhage associated with EVD placement has been documented at rates ranging between 0.7% and 41.0%, influenced by factors such as monitoring time, imaging protocols, and patient-specific variables (97, 100). In contrast, reported rates of infection and hemorrhage associated with ICP monitoring devices were both 1.6% (94). However, Antes et al. observed higher incidences of bleeding and infection in patients monitored with Neurovent-P-tel sensors compared to those using conventional intraparenchymal probes (51).

Another important advantage of long-term ICP monitoring with telemetric sensors is preventing unnecessary invasive interventions when clinical and radiological findings are nonspecific and insufficient to detect ICP changes (27, 41). For example, Korfias et al. stated that patients with normal ICP recordings acquired with telemetric sensors could avoid unnecessary shunt implantations, preventing them from becoming potential “shunt-dependents” for the rest of their lives and mitigating the associated long-term complications (27). Additionally, educating families and patients about ICP monitoring can enhance their role in the treatment process, particularly in pediatric cases (21). This awareness allows patients and their families to make more informed decisions when nonspecific clinical symptoms arise, which has been shown to reduce hospital admission rates, healthcare costs, and anxiety during the treatment process (21, 46).

A noteworthy advantage of the Neurovent-P-tel system is that it requires no calibration and is magnetic resonance imaging (MRI)-compatible at both 1.5 and 3-Tesla (90). The Miethke Sensor Reservoir is also MRI-compatible at the same field strengths, although it can require postimaging recalibration (101). However, further studies are needed to verify the MRI compatibility of other telemetric ICP monitoring devices. Furthermore, for Neurovent-P-tel, ICP values can be reliably obtained either through wound dressings or directly over intact skin, with no significant differences reported. However, clinicians should be aware that the device can be sensitive to ambient temperature changes and performs optimally within a range of 15°C–45°C (54). Additionally, if continuous monitoring is not required for a few hours, the system can be temporarily disengaged by removing the reader and recorder units (54).

Continuous ICP monitoring is particularly advantageous in pediatric patients during long-term follow-up because it can detect subtle increases in ICP that might not be evident through clinical and radiological findings (27, 44). Undetected ICP elevation can harm the developing brain, making continuous monitoring crucial for this vulnerable population (33, 59).

As highlighted in clinical studies, these devices might enhance diagnostic accuracy and improve therapeutic outcomes. Numerous heterogeneous factors that affect shunt drainage, including intraventricular pressure and valve and tube resistances, complicate optimal valve adjustment (32, 102). These problems can lead to complications such as over- and underdrainage, which are associated with various adverse outcomes. To overcome these issues, data acquired from long-term ICP monitoring has been used to fine-tune shunt drainage volumes effectively, a practice successfully demonstrated in previous studies (16, 27, 31, 34, 38, 102). Moreover, considering all these factors, telemetric sensors assist in planning patient-based treatment approaches by assessing preoperative and postoperative ICP changes in relation to circadian and position-related factors, thereby facilitating the selection of appropriate valves and adjusting valve settings accordingly (33, 79, 102).

Additionally, studies have explored the impact of telesensors on service demand and financial outcomes compared with nontelemetric reservoirs (22, 39). Most recently, Pandit et al. demonstrated that using telemetric ICP sensors resulted in significant cost savings and improved resource utilization (22). The study indicates that patients monitored with telesensors required fewer hospital admissions and invasive procedures, such as shunt revisions, further highlighting the potential of these devices to improve both clinical and financial outcomes.

The main challenges associated with telemetric devices include high initial costs that limit accessibility in some healthcare systems, patient discomfort, lack of standardized protocols, and the need for specialized training to interpret data effectively (21, 22, 24). Additionally, intraparenchymal telemetric devices, such as Neurovent-P-tel, have been reported to cause perifocal minor edematous reactions due to factors like initial local tissue trauma during insertion and brain pulsation (27, 51). This edema is generally self-resolving, as reported in a study by Korfias et al. in 2021, but this issue requires further research to understand the long-term implications (27).

Due to substantial heterogeneity in patient populations, study methodologies, and outcome measures across the existing literature, drawing definitive comparisons between telemetric and conventional ICP monitoring techniques remains challenging. Further prospective, standardized investigations are necessary to more accurately assess the relative safety and clinical utility of telemetric ICP monitoring systems. In light of all these factors, the indications for telemetric sensors do not apply to all ICP-related cases; telemetric sensors are best suited to scenarios requiring multiple monitoring sessions or, as noted in numerous studies, select patients who would benefit from long-term monitoring (16, 25, 27, 37, 48, 49).

### 4.6 Future directions

Telemetric ICP monitoring simplifies the management of patients with hydrocephalus who require complex care by providing real-time data on intracranial dynamics, benefiting physicians and patients. However, the literature offers limited indications for different hydrocephalus subtypes. Although telemetric ICP monitoring is recommended for cases that require long-term ICP monitoring, more studies are needed to define its broader applications (16, 58, 61, 63, 102).

In recent years, artificial intelligence algorithms have become increasingly widespread in neurosurgery, paving the way for the potential development of fully integrated systems (103, 104). Future studies should focus on developing a fully integrated system capable of directly measuring ICP and dynamically adjusting the shunt function. Such a system could transform the care of shunt-treated patients by enabling real-time shunt adjustments and minimizing the risks of under- and overdrainage by analyzing complex ICP curves to detect malfunctions.

Although telemetric devices are highly useful and often regarded as problem-solving tools, several technical limitations remain that should be addressed in future studies. For example, the sampling frequency of these devices is significantly lower than that of conventional ICP measurement systems (90). This might potentially reduce the accuracy of the overall recorded data. Additionally, the risk of zero drifting, a gradual deviation of the baseline pressure reading, is a critical concern. Although this issue is also present in conventional systems, it could be more pronounced in telemetric devices due to their longer implantation duration (90). Increasing the sampling frequency in future iterations of these devices could contribute to more objective and accurate measurements.

Another major limitation is the lack of a standardized software platform to analyze the recorded ICP data (90). There is no universally accepted tool for interpreting telemetric readings in a consistent and clinically meaningful manner. Future research should aim to develop such standardized analysis programs, which would enhance data interpretation and clinical decision-making.

Additionally, efforts should focus on enhancing the miniaturization of implanted probes and their long-term biocompatibility to enable true wireless functionality, allowing direct data transmission from the implanted device without requiring interaction with the patient. From the patient's perspective, the potential of telemetric devices to reduce the number of routine medical visits, invasive procedures, and radiation exposure improves patient experience and safety. Although these innovations are hypothesized to yield cost savings in long-term management, more studies are necessary to validate this hypothesis and explore their economic impact in different clinical settings (33).

Also, to address common challenges associated with these probes, specific preventive strategies can be considered. For instance, to mitigate the disconnection issues frequently encountered with some of these devices, more secure anchoring techniques could be developed, and less invasive fixation methods could be explored. These improvements might help prevent accidental disconnections or positional shifts during monitoring.

Although complications such as brain-pulsation-induced injuries or perifocal edema, with a reported incidence of up to 47%, can also occur with conventional ICP monitoring methods, they are typically less prominent because of the short-term nature of these techniques (51). Regarding the safety of telemetric ICP monitoring devices, prospective clinical and biomechanical studies are essential to assess long-term complications, including edematous reactions, infections, and hemorrhages, particularly in pediatric patients (51).

Technical problems related to ICP measurements, such as sensor drift and zero-point variation, must also be addressed. Zero-point variation is a shift in baseline measurements that can compromise reliability, particularly in long-term monitoring. Zero-point drift resulting from material fatigue, fluid ingress into electronics, and temperature variations has historically posed a significant obstacle to the sustained use of telemetric ICP devices (33). Although reengineered designs have mitigated this issue, studies have shown that telemetric devices can deviate by 4 mm Hg from absolute ICP values because of differences in measurement locations and technical factors (16, 33). Ongoing work in entirely passive circuit design could enable recalibration by switched reference signals (105). Integrating more durable materials and dynamic calibration systems could further reduce these challenges, enhance measurement accuracy, improve patient outcomes, and support the broader clinical adoption of telemetric ICP devices.

Numerous studies have highlighted that ICP-guided valve adjustments achieved through telemetric sensors hold significant promise for the future of hydrocephalus management. Although challenges related to high initial costs, uncomfortable devices, and data interpretation remain (22–24), the benefits of continuous monitoring and improved patient outcomes underline telemetry's potential. Telemetry's role in improving outcomes and reducing invasive procedures is transformational in the care of patients with hydrocephalus (16, 25, 48, 49). Addressing these challenges and refining measurement techniques will likely enhance patient outcomes while increasing efficiency in the delivery of healthcare services.

Although our study focuses on direct telemetric monitoring of ICP, various indirect methods are also emerging to predict elevated ICP and delayed cerebral ischemia, particularly through advanced imaging-based assessments (106). Recent efforts have introduced imaging-derived indices to evaluate cerebral hemodynamics and stratify the risks of hydrocephalus, vasospasm, delayed cerebral ischemia, and poor functional outcomes after aneurysmal subarachnoid hemorrhage. One such tool is the cortical vein opacification score, a semi-quantitative CT angiography–based scoring system that assesses cortical venous filling and has shown prognostic value in this patient population (106). This score can serve as a radiologic surrogate of intracranial venous flow patterns. Lower cortical vein opacification scores can reflect delayed cerebral ischemia or suspected ICP elevation, and they could be indirectly associated with an increased risk of hydrocephalus development, although further studies with larger sample sizes are needed to evaluate this association (106). Additional studies are warranted to determine whether such imaging-based tools can complement telemetric ICP monitoring, particularly in diagnostically challenging or equivocal cases of hydrocephalus secondary to aneurysmal subarachnoid hemorrhage.

Lastly, it is worth noting that the existing literature primarily consists of patient cohorts and case reports that combine adult and pediatric populations, limiting understanding of pediatric-specific outcomes. More studies are needed to evaluate the effectiveness of these devices in managing specific patient populations, such as pediatric patients.

## 5 Conclusions

Telemetric ICP monitoring represents a significant advancement in the management of hydrocephalus, addressing the limitations of conventional invasive methods by providing long-term, wireless pressure measurements. This study found growing clinical adoption of these devices. The ability to monitor ICP in real-world conditions has led to improved patient outcomes, fewer unnecessary interventions, and reduced healthcare costs. However, challenges such as sensor drift, standardization of clinical protocols, and cost barriers must be addressed through continued research and technological advancements. Future studies should focus on refining device accuracy, expanding indications for use, and integrating telemetric monitoring into broader neurosurgical practices. As these technologies evolve, they should be expected to transform hydrocephalus management and optimize neurosurgical care.
